# Dynamic presenilin 1 and synaptotagmin 1 interaction modulates exocytosis and amyloid β production

**DOI:** 10.1186/s13024-017-0159-y

**Published:** 2017-02-13

**Authors:** Katarzyna Marta Zoltowska, Masato Maesako, Iryna Lushnikova, Shuko Takeda, Laura J. Keller, Galina Skibo, Bradley T. Hyman, Oksana Berezovska

**Affiliations:** 1 Department of Neurology, MassGeneral Institute for Neurodegenerative Disease, Massachusetts General Hospital, Harvard Medical School, 114 16th Street, Rm. 2006, 02129 Charlestown, MA USA; 2 Department of Cytology, Bogomoletz Institute of Physiology, National Academy of Sciences of Ukraine, 4 Bogomoletz Street, 01024 Kyiv, Ukraine

**Keywords:** Presenilin 1, Synaptotagmin 1, Synapse, Exocytosis, Amyloid β, Alzheimer’s disease

## Abstract

**Background:**

Alzheimer’s disease (AD)-linked protein, presenilin 1 (PS1), is present at the synapse, and the knock-out of presenilin in mice leads to synaptic dysfunction. On the other hand, synaptic activity was shown to influence PS1-dependent generation of distinct amyloid β (Aβ) species. However, the precise nature of these regulations remains unclear. The current study reveals novel role of PS1 at the synapse, and deciphers how PS1 and synaptic vesicle-associated protein, synaptotagmin 1 (Syt1) modulate each other functions in neurons via direct activity-triggered interaction. Additionally, the therapeutic potential of fostering PS1-Syt1 binding is investigated as a synapse-specific strategy for AD prevention.

**Methods:**

PS1-based cell-permeable peptide targeting PS1-Syt1 binding site was designed to inhibit PS1-Syt1 interaction in neurons. PS1 conformation, synaptic vesicle exocytosis and trafficking were assayed by fluorescence lifetime imaging microscopy (FLIM), glutamate release/synaptopHluorin assay, and fluorescence recovery after photobleaching, respectively. Syt1 level and interaction with PS1 in control and sporadic AD brains were determined by immunohistochemistry and FLIM. AAV-mediated delivery of Syt1 into mouse hippocampi was used to investigate the therapeutic potential of strengthening PS1-Syt1 binding in vivo. Statistical significance was determined using two-tailed unpaired Student’s t-test, Mann-Whitney’s *U*-test or two-way ANOVA followed by a Bonferroni’s post-test.

**Results:**

We demonstrate that targeted inhibition of the PS1-Syt1 binding in neurons, without changing the proteins’ expression level, triggers “pathogenic” conformational shift of PS1, and consequent increase in the Aβ42/40 ratio. Moreover, our data indicate that PS1, by binding directly to Syt1, regulates synaptic vesicle trafficking and facilitates exocytosis and neurotransmitter release. Analysis of human brain tissue revealed that not only Syt1 levels but also interactions between remaining Syt1 and PS1 are diminished in sporadic AD. On the other hand, overexpression of Syt1 in mouse hippocampi was found to potentiate PS1-Syt1 binding and promote “protective” PS1 conformation.

**Conclusions:**

The study reports novel functions of PS1 and Syt1 at the synapse, and demonstrates the importance of PS1-Syt1 binding for exocytosis and safeguarding PS1 conformation. It suggests that reduction in the Syt1 level and PS1-Syt1 interactions in AD brain may present molecular underpinning of the pathogenic PS1 conformation, increased Aβ42/40 ratio, and impaired exocytosis.

**Electronic supplementary material:**

The online version of this article (doi:10.1186/s13024-017-0159-y) contains supplementary material, which is available to authorized users.

## Background

Aberrant amyloid β (Aβ) generation resulting in increased Aβ42/total ratio, and loss of cortical and hippocampal synapses have been established as important contributors to Alzheimer’s disease (AD) pathophysiology [1–8]. However, amyloid deposition and reduced levels of synaptic proteins found in postmortem human brain tissue do not reveal the early stage molecular events preceding the observed pathology, and do not allow to determine dynamic functional relationship between the synaptic impairments and Aβ generating machinery.

The amyloid precursor protein (APP) and its processing enzymes, β-secretase 1 (BACE1) and presenilin 1 (PS1)/γ-secretase, are abundantly present at the synaptic terminals [9–12], enabling local production of Aβ. The generation of the longer Aβ42/43 species readily forming toxic oligomers is influenced by the PS1/γ-secretase conformational state [13–20]. Fluorescence lifetime imaging microscopy (FLIM) and real-time Förster resonance energy transfer (FRET) assays of the PS1 domain arrangement demonstrated that PS1 exists in a dynamic equilibrium of conformational states. Although the assays do not address the detailed structural rearrangements within the PS1 molecule, they can reliably distinguish between the normal, so-called “open” and pathogenic, named “closed” PS1 conformations. These are characterized by greater and smaller distance, respectively, between the fluorophores labeling different PS1 domains. “Open” and “closed” PS1 conformations correspond to lower and higher ratio of the Aβ42 to Aβ40 peptides, respectively [12, 15, 21, 22]. However, the precise molecular mechanisms altering the arrangement of the PS1 domains in neurons remain unknown.

Although the role of PS1 in Aβ generation has received the greatest attention, presenilin knockout studies have suggested that PS1 may also be involved in neurotransmission [23, 24]. However, the exact role of the “undisturbed” PS1, expressed at the physiological level, and the specific molecular mechanism of its involvement in synaptic vesicle release remain unexplored.

It has recently been reported that both APP [25, 26] and PS1 interact with the pre-synaptic protein synaptotagmin 1 (Syt1) [12], a calcium sensor in synaptic vesicle exocytosis and neurotransmitter release [27–29]. Moreover, knock-down of Syt1 in PC12 cell line was found to alter APP processing and reduce Aβ generation [12, 25]. However, Syt1 knock-down also impaired subcellular compartmentalization of PS1 and BACE1, resulting in decreased maturation and stability of BACE1, and diminished γ-secretase activity [12, 25]. Therefore, the significance of the direct PS1-Syt1 binding per se, as well as functional outcomes of the disrupted PS1-Syt1 interaction for synaptic physiology and/or their relevance to AD pathology remain unknown.

In the current study we investigate in detail the functional interplay between PS1 and Syt1 by selectively targeting the PS1-Syt1 binding using PS1-based cell-permeable peptide corresponding to the PS1-Syt1 interaction site, without altering the proteins’ expression level. Using complementary cell/molecular biology approaches and functional assays, we present evidence that calcium influx-induced binding between PS1 and Syt1 is essential for regulation of the synaptic vesicle trafficking along neuronal processes, promotion of exocytosis and neurotransmitter release. Moreover, we provide direct evidence that Syt1 binding to PS1 is important for maintenance of the protective PS1 conformation in neurons, and for determination of the local, synaptic pool of Aβ species. Finally, we demonstrate that not only Syt1 levels but also proximity between the remaining Syt1 and PS1 is decreased in sporadic AD brain, and that overexpression of Syt1 in vivo has potential to promote PS1-Syt1 interaction, and to stabilize the protective, “open” PS1 conformation.

## Methods

### Human brain tissue

The medial temporal cortex of the sporadic Alzheimer’s disease (sAD) patients (92.25 ± 4.52 years old) with neuropathologically confirmed Braak V-VI stage, and age- (90.75 ± 1.60 years old) and post mortem interval-matched control tissue was obtained from the Massachusetts Alzheimer’s Disease Research Center Brain Bank. For the immunohistochemical analysis, medial temporal lobe specimens were fixed for 48 h with 4% paraformaldehyde (PFA) and cut into 50 μm coronal sections using a sliding freezing microtome (Leica SM 2000R, Bannockburn, IL).

### Animal research

Syt1-V5 expression in vivo in hippocampi of wild type male C57BL/6 mice was achieved using stereotaxic injection (stereotaxic coordinates from bregma: 3.1 mm posterior; 2.8 mm lateral and 2.5 mm depth from brain surface) of adeno-associated viruses serotype 2/8 (AAV2/8) carrying hSyn. Syt1-V5 or control, empty, expression plasmids. One month after the AAV2/8 injection, the mice were sacrificed.

The mice were euthanized using CO_2_ asphyxiation and perfused intracardially with phosphate-buffered saline (PBS). The brains were dissected, fixed by immersion in 4% PFA, 15% glycerol (Sigma-Aldrich, Saint Louis, MO) in PBS and cryoprotected using 30% glycerol in PBS. Prior to immunostaining, the brains were cut on freezing sliding microtome (Leica SM 2000R, Bannockburn, IL) into 35 μm-thick coronal sections.

### Cell culture

Rat pheochromacytoma cell line (PC12) was a kind gift from Dr. Amy B. Harkins (St. Louis University School of Medicine, St. Louis, MO) [30]. The PC12 cells were cultured in RPMI Medium 1640 supplemented with 10% heat-inactivated horse serum, 5% fetal bovine serum (FBS) (ThermoScientific, Waltham, MA) in a 37 °C, 5% CO_2_ incubator. Transfections were performed using lipofectamine 3000 (ThermoScientific, Waltham, MA) according manufacturer’s protocol.

Mouse embryonic fibroblasts (MEF) with *PSEN1* and *PSEN2* knock-out (PS DKO) and MEF PS DKO stably expressing PS1 wt or PS1 ∆e9 were kind gifts from Dr. Bart De Strooper [31]. The cells were maintained in OptiMEM supplemented with 5% fetal bovine serum (FBS) (ThermoScientific, Waltham, MA) in a 37 °C, 5% CO_2_ incubator. Transfections were performed using lipofectamine LTX with Plus reagent (ThermoScientific, Waltham, MA) according manufacturer’s protocol.

Mixed cortical primary neurons from 16 to 18 embryonic-day-old embryos were enzymatically dissociated using papain dissociation system (Worthington Biochemical Corporation, Lakewood, NJ). The neuronal cultures were maintained in Neurobasal medium supplemented with 2% B27 supplement, 1% GlutaMax, and 1% penicilin/streptomycin mix (ThermoScientific, Waltham, MA) in a 37 °C, 5% CO_2_ incubator. The neurons were transfected using lipofectamine 2000 (ThermoScientific, Waltham, MA) following the supplier’s protocol.

### Chemicals and treatments

Calcium influx was induced by 15-min application of 50 mM KCl (Sigma-Aldrich, Saint Louis, MO) for neurons and PC12 cell line, or of 5 μM A23187 calcium ionophore (Sigma-Aldrich, Saint Louis, MO) for MEFs.

PS1-Syt1 interactions were blocked by incubating primary neurons for 2 h at 37 °C with 5 μM of cell-permeable peptide (CPP), we named PS1-LNT. The PS1-LNT was obtained by fusing 47–57 amino acids (aa) from HIV1 TAT protein (YGRKKRRQRRR) with the N-terminal portion of the cytosolic PS1 loop domain through a GGG linker. A peptide comprising 47–57 aa from HIV1 TAT fused to a scramble sequence ENSFRFLADIFPAKAFPVRFE through a GGG linker was used as a negative control. The peptides were synthesized at the MGH peptide/protein core facility https://researchcores.partners.org/pepcor/about.

### Expression plasmids

Human wild type (wt) presenilin 1 (PS1) was cloned into pcDNA™3.1. (+) (ThermoScientific, Waltham, MA). The PS1 sequence was tagged with an N-terminal FLAG and His tags to facilitate detection of exogenous versus endogenous PS1.

The His-FLAG-huPS1 construct (PS1 del265-279) was created by introducing 15 aa deletion within the huPS1 sequence using a QuickChange site-directed mutagenesis kit (Stratagene, La Jolla, CA) according to the manufacturer’s recommendation.

Human wt synaptotagmin 1 (Syt1) was cloned into pcDNA™ 6 V5 Myc expression vector (ThermoScientific, Waltham, MA).

Plasmids encoding vesicular glutamate transporter 1 (vGlut1) fused with pH-sensitive GFP (synaptophluorin (SypHy)), eGFP-tagged synaptophysin (eGFP-Syp) and eGFP-tagged tubulin (eGFP-Tub) were kind gifts from Dr Pamela McLean (Mayo Clinic, Jacksonville, FL).

### Cytotoxicity assay

Cytotoxicity was analyzed using lactate dehydrogenase (LDH) cytotoxicity assay (Roche, Indianapolis, IN). Briefly, conditioned medium was collected from the respective wells, mixed with the assay solution, incubated for 20 min in the dark, and the absorbance at 490 nm was measured using a spectrophotometer. For a positive control, cells were incubated for 45 min at 37 °C with 1% Triton X (TX)-100.

### ELISA for Aβ40 and Aβ42

Intracellular or secreted level of Aβ was quantified using human/rat Aβ40 and Aβ42 (high-sensitive) enzyme-linked immunosorbent assay (ELISA) kits (Wako, Japan) according to the manufacturer’s protocol. The Aβ levels detected in the conditioned medium or cell lysates (determined in [pmol]), were normalized to the total amount of protein extracted from the respective cells (quantified in [g]) using BCA protein assay (Pierce, Rockford, IL).

### Glutamate release assay

Glutamate release was stimulated by application of 50 mM KCl in Hank’s balanced salt solution after 2-h pre-treatment with scramble or PS1-LNT peptides. The glutamate uptake was inhibited by the addition of *DL*-threo-β-benzyloxyaspartate (DL-TBOA), an excitatory amino acid transporters 1 and 2 (EAAT1, EAAT2) inhibitor [32]. The level of glutamate in the conditioned medium was determined using Amplex Glutamate assay (ThermoScientific, Waltham, MA) according to the manufacturer’s protocol. Overnight pre-treatment with 37 nM tetanus toxin (TeTx) was used as a control.

### NanoOrange protein quantitation

The total amount of protein in the conditioned medium collected from neurons pre-treated for 2 h with scramble or PS1-LNT peptides, and stimulated for 15 min with 50 mM KCl was determined using NanoOrange protein quantitation assay (ThermoScientific, Waltham, MA), according the manufacturer’s protocol. Briefly, the serum-free conditioned medium was diluted 100 times in NanoOrange reagent, and the mixtures were heated at 95 °C for 10 min protected from light. The samples were cooled down to room temperature, and the fluorescence, corresponding to the total amount of protein in the samples, was measured using Wallac Victor 2 plate reader (PerkinElmer, Waltham, MA) with filters and settings allowing excitation at 485 nm and capturing the emission at 590 nm. Standard curve was generated by serial dilution of the bovine serum albumin (BSA) in the NanoOrange reagent.

### Exocytosis assay

The exocytosis was monitored using a previously established protocol [33, 34]. Briefly, the primary neurons were transfected with a construct encoding a pH-sensitive reporter of synaptic vesicle exocytosis, synaptophluorin (SypHy). The acidic pH of the synaptic vesicles results in quenching of the SypHy fluorescence. During the exocytosis event the pH-sensor is exposed to the extracellular neutral pH, which results in the dequenching of the SypHy signal. The SypHy was excited using 488 nm Argon laser at low power, 4–6% to reduce photobleaching and phototoxicity, and the fluorescence was monitored at 63x magnification using time-lapse recording with 3 s interval on Zeiss 510 Meta laser scanning confocal microscope equipped with ZEN 2009 software. First, five frames were collected before the stimulus to determine the baseline SypHy fluorescence in cells pre-treated for 2 h with the scramble or PS1-LNT peptides at 5 μM concentration. Then exocytosis was triggered by 50 mM KCl stimulation, and the subsequent frames were acquired immediately following the KCl application. The laser intensity and photomultiplier parameters (PMIs) were adjusted to avoid saturation of the signal on both pre- and post-stimulus images. The fluorescence intensity of the SypHy pre- and post-stimulus was analyzed using ImageJ 1.46c software. The region of interests (ROIs) were selected around the spatially resolved SypHy-positive puncta corresponding to synaptic terminals.

### Fluorescence recovery after photobleaching (FRAP)

The 12-14 DIV neurons were transfected with plasmids encoding eGFP-synaptophysin (eGFP-Syp) as a fluorescent reporter of synaptic vesicles or eGFP-tubulin (eGFP-Tub) as a control. For the imaging the culture medium was substituted with Hank’s balanced salt solution (HBSS) (ThermoScientific, Waltham, MA), and the cell culture dishes were placed on an imaging stage in the microscope environmental chamber (37 °C, 5% CO_2_). The eGFP was excited using 488 nm Argon laser at low power (2–4%) to avoid photobleaching during the time-lapse imaging; the emitted light was passed through a band pass emission filter. Five pre-bleach images at 63x magnification with 2x zoom were recorded to determine the baseline fluorescence intensity of the target protein. Then, the eGFP-Syp or eGFP-Tub fluorescence was photobleached within the selected ROIs using 75 iterations with 100% 488 nm Argon laser, and the imaging was continued for 90 s using time-lapse recording with 1-s intervals. Three ROIs were recorded and analyzed: bleached, control (not bleached), and background. The fluorescence intensities of the selected ROIs were measured using ImageJ 1.46c software.

The recorded data were analyzed with Microsoft Excel 2007 and GraphPad Prism 5 software. Following the background subtraction, the intensity of the bleached ROI was normalized to its initial pre-bleach intensity (calculated as an average of the 5 pre-bleach frames) and to the intensity of the control, not bleached region. Then the data were further normalized in respect to the intensities immediately after bleaching (value = 0, full scale normalization). In order to determine the statistical difference between the conditions, the mean fluorescence recovery 10 s post-bleach was compared using unpaired Student’s t-test.

### Analysis of the spine density and spine morphology

Mouse primary neurons at 12–14 DIV were transfected with pcDNA-eGFP. Two days post-transfection the neurons were pre-treated for 2 h with scramble or PS1-LNT peptides and depolarized for 15 min with 50 mM KCl. The spine density and morphology were examined using the LSM 510 Zeiss confocal microscope equipped with the 488 nm excitation laser. The images were captured at 63x magnification, and consisted of a *z*-stack of pictures taken at 0.5 μm intervals, and then projected into one image. The images were analyzed using NeuronStudio software [35]. The spine density was calculated by dividing the number of spines by the length of the corresponding neurite. In addition, the spines were categorized into three morphological classes: mushroom, stubby and thin using an algorithm implemented in the NeuronStudio application. 30 neurites per condition from eight independent experiments were analyzed.

### Immunoprecipitation (IP)

Cells were lysed in 1% 3-[(3-cholamidopropyl) dimethylammonio]-2-hydroxy-1-propanesulfonate (CHAPS) buffer (50 mM HEPES, 100 mM NaCl, pH 7.4 and 1% CHAPS) supplemented with HALT protease and phosphatase inhibitor cocktails (ThermoScientific, Waltham, MA) by pipetting up-and-down, passing through a 37-gauge needle and rotating for 1 h at 4 °C. The lysates were centrifuged at 14,000xg for 15 min and the supernatants were collected for the IP. Total protein in the samples was measured using ThermoScientific Pierce BCA Protein Assay (ThermoScientific, Waltham, MA). The aliquots of the supernatant containing equal protein amount were incubated with 3 μg of the respective antibody or normal IgG, as a negative control, with end-over-end rotation over night at 4 °C. Subsequently, 30 μl of Protein G Dynabeads (ThermoScientific, Waltham, MA) were added to the samples and incubated with end-over-end rotation for 10 min at room temperature. The beads were collected using a magnetic tube rack, washed twice with the 1% CHAPS buffer and once with the wash buffer containing 50 mM HEPES, 100 mM NaCl, pH7.4. The protein was eluted by 5-min boiling in 25 μl 2x LDS, 1x reducing agent buffer (ThermoScientific, Waltham, MA).

### Immobilized metal affinity chromatography (IMAC)

IMAC technique was used to pull down His-PS1 and endogenous Syt1 complexes from PC12 cells transfected with His-tagged PS1 wt or PS1 del265-279. Briefly, 24 h following the transfection the cells were stimulated for 15 min with 50 mM KCl, harvested and lysed in a buffer containing 50 mM HEPES, 100 mM NaCl, protease inhibitors (Roche, Indianapolis, Indiana) and 1% CHAPS. The lysates were incubated for 1 h at 4 °C and centrifuged at 14,000xg for 15 min to collect the soluble fractions. Protein concentration in each sample was determined using a BCA assay (ThermoScientific, Waltham, MA) according the manufacturer’s recommendations. The samples with equal amount of protein were supplemented with 20 mM imidazole to reduce non-specific binding, and were incubated for 2 min with 30 μl MagneHis Ni-Particles (Promega, Madison, WI). Following the incubation, the beads were washed 3 times with the lysis buffer, and the protein was eluted with the elution buffer provided with the MagneHis Ni-Particles, containing 500 mM imidazole.

### In vitro PS1-Syt1 binding assay

Syt1 tagged with V5 and His was overexpressed in PS DKO MEF cells. The protein was extracted from the cells using a lysis buffer containing 50 mM HEPES, 100 mM NaCl, protease inhibitors (Roche, Indianapolis, Indiana) and 1% TX-100, and was immobilized on the MagneHis Ni-Particles (Promega, Madison, WI) by 2-min incubation in the presence of 20 mM imidazole to reduce non-specific binding. Subsequently, 30 μl of the Syt1-V5-His bound beads were added to the mouse brain lysates containing 2 mg of total protein and prepared in a buffer containing 50 mM HEPES, 100 mM NaCl, protease inhibitors (Roche, Indianapolis, Indiana), 1% CHAPS, 20 mM imidazole, 2 mM CaCl_2_. 100 μg of PS1-LNT or scramble, as a negative control, peptides were added to the samples. Additional negative controls included Syt1-V5-His bound beads incubated with the lysis buffer only, or empty beads incubated with the mouse brain lysates. The mixtures were incubated overnight at 4 °C with end-over-end rotation. Then, the beads were washed 3 times with the 1% CHAPS-based lysis buffer, and the attached protein was eluted with the elution buffer provided with the MagneHis Ni-Particles, containing 500 mM imidazole.

### Western blotting

The proteins were resolved by electrophoresis on 4–12% Bis-Tris NuPage polyacrylamide gels (ThermoScientific, Waltham, MA) and transferred to nitrocellulose membranes (GE Healthcare Lifesciences, Pittsburgh, PA) using the BioRad system. Proteins were detected by immunoblotting with specific primary and corresponding IRdye680/800- or HRP-conjugated secondary antibodies. The membranes were developed using Odyssey Infrared Imaging System (Li-COR, Lincoln, NE) or ECL Western Blotting Substrate (Pierce, Rockford, IL), respectively. The exposure times were adjusted to avoid signal saturation. The relative amounts of the proteins were measured by densitometry using ImageStudio Lite Ver 5.2 or ImageJ 1.46c software, respectively.

### Immunocytochemistry (ICC) and immunohistochemistry (IHC)

Cultured cells or brain tissue slices were fixed with 4% PFA, permeabilized using 0.1% TX-100 or 0.4% TX-100, respectively, and non-specific binding of the antibodies was blocked by incubation with 1.5% normal donkey serum (Jackson ImmunoResearch Labs, West Grove, PA). The permeabilized and blocked samples were incubated with primary antibodies overnight at 4 °C. Excess antibodies were washed off and the samples were incubated with corresponding Alexa Fluor 488- or Cy3-conjugated secondary antibodies for 1 h at room temperature. To visualize actin cytoskeleton, the cells were incubated with fluorophore-conjugated phalloidin (ThermoScientific, Waltham, MA) according to the manufacturer’s protocol. The medium was supplemented with 4′, 6-diamidino-2-phenylindole dihydrochloride (DAPI) when visualization of nuclei was required. The slides were mounted with VectaShield mounting medium (Vector Laboratories, Inc., Burlingame, CA).

### Antibodies

The following primary antibodies were used: anti-APP CT (A8717, Sigma-Aldrich, St. Louis, MO); anti-β-amyloid, 17–24 (clone 4G8) (800701, BioLegend, San Diego, CA); anti-PS1 NT raised against the N terminus of PS1 (APS11) (ab15456, Abcam, Cambridge, MA); anti-PS1 CT raised against the C-terminus of PS1 (mAb5643, Cell Signaling Technology, Danvers, MA); anti-PS1 loop raised against the loop domain between transmembrane domains 6 and 7 of PS1 (E2000Y) (ab76083, Abcam, Cambridge, MA); anti-Syt1 (AB5600, Millipore, Temecula, CA); anti-Gapdh (mAb2118, Cell Signaling Technology, Danvers, MA); anti-MAP2 (ab5392, Abcam, Cambridge, MA); anti-His (ab18184, Abcam, Cambridge, MA); anti-V5 (ab9116, Abcam, Cambridge) and anti-FLAG M2 (F1804, Sigma-Aldrich, St. Louis, MO). Alexa Fluor 488 (ThermoScientific, Waltham, MA) and Cy3-labeled corresponding secondary antibodies (Jackson ImmunoResearch, West Grove, PA) were used for confocal microscopy imaging, and IRDye680/800- (Li-COR, Lincoln, NE) or HRP- (Jackson ImmunoResearch, West Grove, PA) conjugated ones were used for western blotting.

### Fluorescent lifetime imaging microscopy (FLIM)

The proximity between PS1 and Syt1 or PS1 NT and PS1 loop domain was evaluated by a previously validated Förster Resonance Energy Transfer (FRET)-based fluorescence lifetime imaging microscopy (FLIM) assay [22, 36, 37]. The samples were immunostained with the following pairs of primary antibodies: a) anti-PS1 NT (APS11) (ab15456, Abcam, Cambridge, MA) and anti-Syt1 (AB5600, Millipore, Temecula, CA) or anti-FLAG M2 (F1804, Sigma-Aldrich, St. Louis, MO) and anti-Syt1 for analysis of PS1-Syt1 interaction; b) anti-PS1 NT (APS11) and anti-PS1 loop (EP2000Y) (ab76083, Abcam, Cambridge, MA) for the analysis of PS1 conformation. Alexa Fluor 488 (AF488) and Cy3-labeled corresponding secondary antibodies were used as the donor and acceptor fluorophores, respectively. AF488 donor fluorophore was excited using pulsing Chameleon Ti:Sapphire laser (Coherent Inc., Santa Clara, CA) (two-photon excitation at 780 nm or 800 nm wavelength, respectively). The baseline lifetime (*t*1) of the AF488 donor fluorophore in the absence of the Cy3 acceptor was used as a negative control. The donor lifetimes were determined using a high-speed photomultiplier tube (MCP R3809; Hamamatsu, Bridgewater, NJ) and a fast time-correlated single-photon counting acquisition board (SPC-830; Becker & Hickl, Berlin, Germany). Bright fluorophores were chosen, and prolonged image acquisition at low laser power was applied in order to acquire sufficient number of photons for accurate curve fitting, without photobleaching of the samples. The lack of photobleaching was established by the lack of noticeable decrease in the count rate during acquisition time, and was confirmed by analysis of the fluorescence intensity in pre- and post-acquisition confocal images. Typically, 1,000–5,000 photons per analyzed pixel (within the selected intensity-thresholded region of interest (ROI)) were acquired. If necessary, binning was applied to increase the signal to noise ratio. The data were analyzed using SPCImage software (Becker & Hickl, Berlin, Germany). The analysis was performed on a cell-by-cell basis, and the threshold was adjusted in order to exclude the low intensity background fluorescence. The ROIs were selected by outlining the neurons, and the average fluorescence lifetimes per neuron were determined. Two analysis modes were applied: two component analysis for cells in vitro and mouse tissue and three component analysis for human brain tissue to account for the presence of autofluorescence, as described previously [38]. The lifetimes of the “non-FRET” donor (*t*_1_), and if applicable of the autofluorescence (*t*_3_), were “fixed” and the remaining lifetime reflecting the presence of FRET was calculated by the system as the *t*_2_ value. This approach allows the exclusion of the “non-FRETing” components, and thus reliable quantification of the relative distances between the fluorophores, irrespectively of their absolute amounts. Moreover, it significantly improves the accuracy of the multicomponent analysis [39]. The fluorescence lifetime of the “non-FRETing” donor, determined independently in every single experiment was ~2.5 ns. Of note, the 4.1 ns has been reported for the unconjugated Alexa Fluor 488 dye. However, conjugation of the dye to the antibody results in the reduction in its fluorescence lifetime [40]. To calculate %E_FRET_ the following equation was used: %E_FRET_ = 100*(*t*_1_-*t*_2_)/*t*_1_.

The calculated lifetimes *t*_*2*_ were displayed on a 128×128 pixel matrix to create the pseudocoloured images where green-to-red pixels represent shorter lifetimes, indicative of short distance between the fluorophores, whereas blue pixels correspond to longer lifetimes, indicative of a greater distance between the donor and the acceptor. In the images, the lifetimes were weighted by intensity, and a threshold was adjusted to exclude dim pixels. Importantly, all the analyses and the display parameters were kept constant in each experiment, providing the most accurate quantification of the acquired data.

### Electron microscopy

Electron microscopy was applied to analyze the size and distribution of the synaptic vesicles in mouse primary neurons in vitro. The 12-14 DIV neuronal cultures were fixed with ice-cold 2% PFA and 0.2% glutaraldehyde (Sigma-Aldrich, St. Louis, MO) for 1 h at 4 °C. Then the cells were washed three times with 0.1M phosphate buffer pH 7.4 (PB), stained with 1% OsO_4_ in 0.1 M PB for 1 h, dehydrated in graded ethanol series and flat-embedded in EPON (Fluka). Ultrathin (50–60 nm thick) sections were cut with an ultratome (LKB 8800, Sweden), stained for 20 min with 5% uranyl acetate and 30 s in lead citrate, and examined using a JEM100-CX transmission electron microscope (Japan). The images, taken at magnifications of x19,000, were scanned with a flatbed scanner at 600 dpi and 256 gray levels. Quantitative image analysis was carried out using the ImageJ 1.46c software.

### Statistics

Statistics were calculated with Microsoft Office Excel 2007. The graphs were prepared with GraphPad® Prism 5 (GraphPad Prism Software inc., La Jolla, CA). Gaussian distribution of the data was determined using D’Agostino & Pearson omnibus normality test. The F statistics were calculated to determine the variance equality. Subsequently, a standard two-tailed unpaired Student’s t-test or Welch t-test was calculated for the normally distributed data and Mann-Whitney’s *U*-test for the data, which did not meet the latter criterion. Glutamate release, cytotoxicity and frequency distribution of synaptic vesicles data were analyzed using two-way ANOVA followed by a Bonferroni’s post-test. A *p*-value of <0.05 was considered a predetermined threshold for statistical significance.

## Results

### Peptide corresponding to the N-terminal fragment of the L6-7 domain of PS1 inhibits PS1-Syt1 interaction

**Fig. 1:**
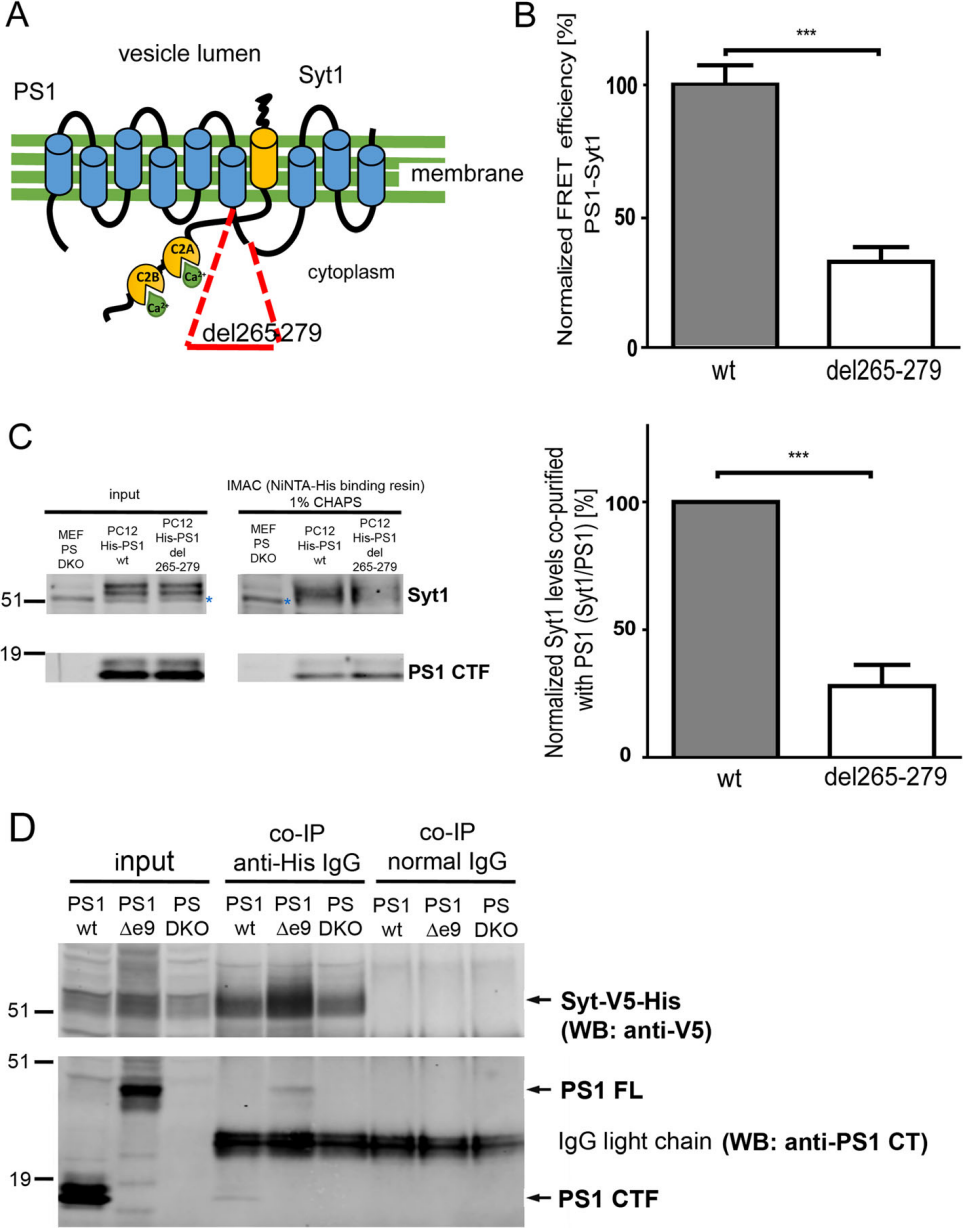
Determination of Syt1-interacting region within PS1 sequence. **a** Schematic presents the PS1-Syt1 complex and the 15-amino acid deletion within the PS1 L6-7 sequence (del265-279). **b** Deletion within the L6-7 domain of PS1 inhibits PS1-Syt1 interaction. PC12 cells transfected with FLAG-tagged wild type (wt) or del265-279 PS1 were immunostained with anti-FLAG and anti-Syt1 antibodies, followed by fluorescently conjugated secondary antibodies, and analyzed by FLIM. The graph shows normalized FRET efficiency between fluorescently labeled endogenous Syt1 and overexpressed PS1 in PC12 cells stimulated for 15 min with 50 mM KCl. The data are presented as mean ± SEM, *n* = 38 for PS1 wt, and *n* = 33 for PS1 del265-279, *n* = total number of cells analyzed in 3 independent experiments. Statistical significance was determined using two-tailed unpaired Student’s t-test, ***p < 0.001. The fluorescence lifetimes and corresponding FRET efficiency values are specified in the Additional file 1. **c** Syt1 co-purifies more efficiently with His-PS1 wt than with His-PS1 del265-279. Overexpressed His-tagged PS1 and endogenous Syt1 complexes extracted from PC12 cells following 15-min 50 mM KCl application were purified using immobilized metal affinity chromatography (IMAC) and analyzed by western blotting; unspecific bands are marked with blue asterisks. PS double knock-out (PS DKO) mouse embryonic fibroblasts (MEF) cells were used as a negative control. The adjacent graph presents quantification of the Syt1 levels co-purified with PS1. The data are presented as mean ± SEM, *n* = 3; two-tailed unpaired Student’s t-test, ***p < 0.001. **d** PS1 with the deleted exon 9 (PS1Δe9) interacts with Syt1. PS DKO and PS DKO cells stably expressing PS1 wt or PS1Δe9 were transiently transfected with His-V5-Syt1 and incubated for 15 min with 5 μM A23187 calcium ionophore. Anti-His antibody or normal mouse IgG as a control were used for the immunoprecipitation, and anti-V5 and anti-PS1 CT antibodies were applied for the immunodetection. The experiment was performed three times

Our previous data suggest that the interaction site between presenilin 1 (PS1) and synaptotagmin 1 (Syt1) is potentially located within the N-terminal part of the PS1 cytosolic loop domain between 6^th^ and 7^th^ transmembrane helices (L6-7) [12]. First, to confirm that this region is indeed critical for the PS1-Syt1 binding, fifteen amino acid deletion (del265-279) was introduced within the PS1 sequence (Fig. 1a). The construct was expressed in PC12 cells, and the proximity between PS1 and Syt1 was analyzed using antibody-based fluorescence lifetime imaging microscopy (FLIM) assay in 15-min 50 mM KCl stimulated cells. N-terminal PS1 FLAG-tag was used for PS1 detection to selectively visualize exogenous mutant PS1. Reduced Förster resonance energy transfer (FRET) efficiency indicates that the PS1 deletion mutant shows reduced binding to Syt1, as compared to the wild type (wt) PS1 (Fig. 1b and Additional file 1), supporting the importance of the PS1 aa 265-279 for PS1-Syt1 interaction.

These findings were further validated by a complementary approach: immobilized metal affinity chromatography (IMAC), designed for selective isolation of the His-tagged proteins. PC12 cells were transiently transfected with His-tagged wt or del265-279 mutant PS1, stimulated for 15 min with 50 mM KCl, and His-PS1 and endogenous Syt1 complexes were analyzed. We found that deletion of the amino acids 265–279 within the PS1 sequence significantly inhibits the PS1-Syt1 binding (Fig. 1c).

Of note, the PS1-Syt1 interaction is not dependent on the PS1 endoproteolysis, as demonstrated by successful pull-down of His-V5-tagged Syt1-PS1 wt as well as His-V5-Syt1-PS1∆e9, lacking the endoproteolysis site, complexes from PS double knock-out mouse embryonic fibroblasts (PS DKO MEF) stably expressing respective PS1 variants and transiently transfected with His-V5-Syt1 (Fig. 1d).

**Fig. 2:**
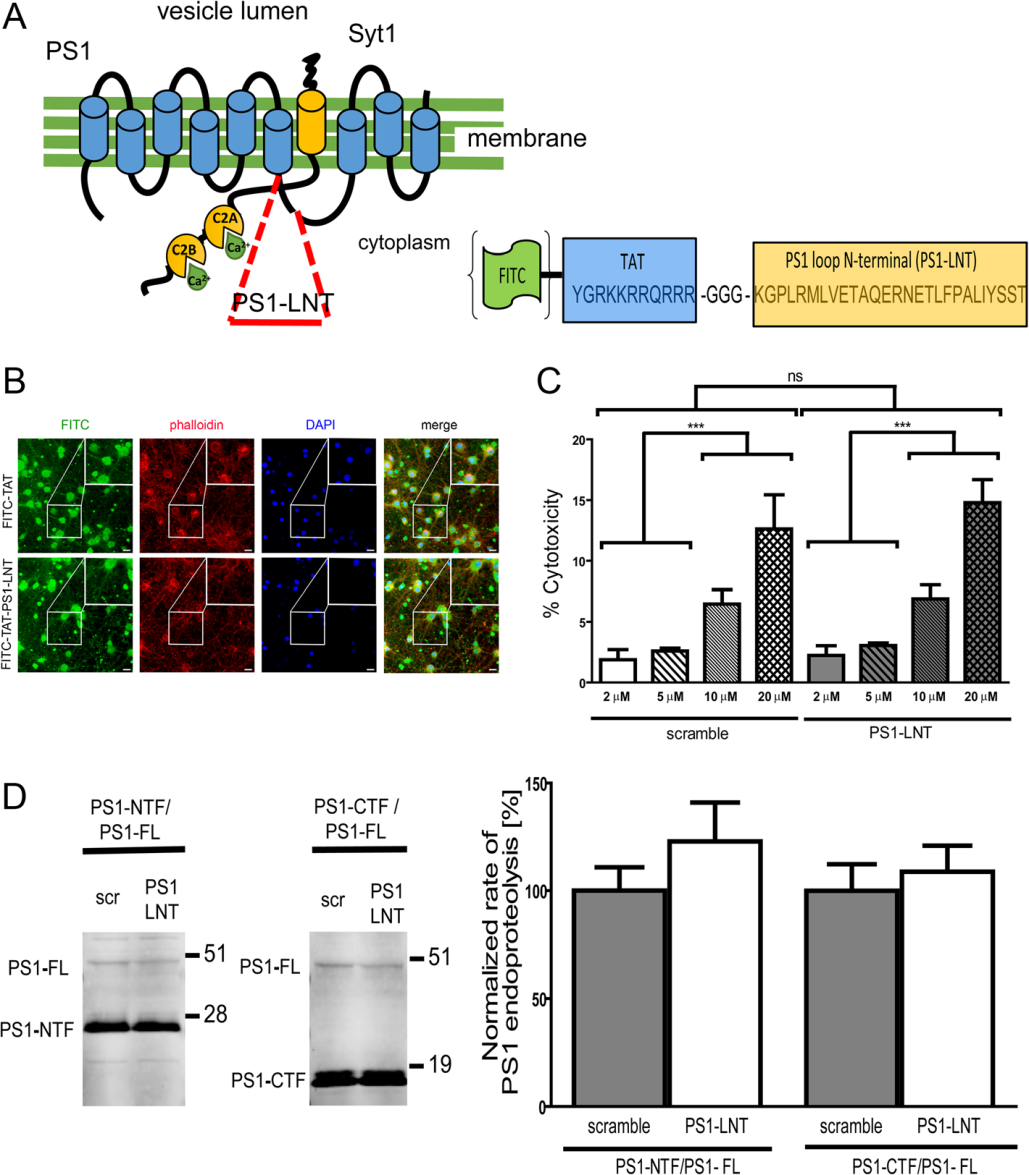
Design of the cell-permeable peptide and its application in primary neurons. **a** Schematic representation of the PS1-Syt1 complex. The PS1 fragment used for the design of the cell-permeable peptide to inhibit the PS1-Syt1 interaction is indicated in red. The adjacent image presents the fusion of the HIV1 TAT domain with the respective PS1 fragment. For initial experiments the peptide was conjugated with fluorescein (FITC) to visualize intracellular delivery. Scramble peptide was used as a control. **b** Representative images demonstrate successful delivery of 5 μM TAT-FITC and TAT-PS1-LNT-FITC (green) peptides to the primary neurons after two-hour incubation. The cells were counterstained with phalloidin (red) and DAPI (blue) to visualize the actin cytoskeleton and the nuclei, respectively; scale bar 10 μm; the magnified inserts demonstrate efficient entry of the FITC-labeled peptides into neuronal cell bodies and processes. **c** Analysis of the cytotoxicity of the scramble and PS1-LNT peptides in neurons after two-hour incubation at the respective concentrations. The % cytotoxicity was determined using lactase dehydrogenase (LDH) activity assay. The data are normalized to the 1% TX-100 condition, considered to cause 100% toxicity. There is negligible toxicity of scramble and PS1-LNT peptides used at 2 μM and 5 μM final concentrations. The data are presented as mean ± SEM, *n* = 37 for 5 μM, *n* = 13 for 10 μM and *n* = 7 for the remaining conditions. Statistical significance was determined using two-tailed unpaired Student’s t-test, ***p < 0.001. **d** Representative western blot shows efficient endoproteolysis of the full length PS1 (PS1-FL) to yield PS1-NTF and PS1-CTF in control and PS1-LNT pre-treated neurons. The graph presents quantification of the optical density of the respective bands. The ratio between PS1-CTF or PS1-NTF and PS1-FL was used to estimate endoproteolysis efficiency. The data are presented as mean ± SEM, *n* = 7. Statistical significance was determined using two-tailed unpaired Student’s t-test, ns *p* > 0.05

Based on these data, we designed a peptide corresponding to the N-terminus of the PS1 loop domain (PS1-LNT) to inhibit the PS1-Syt1 binding. The fragment was fused with positively charged TAT HIV1 epitope to enable cell permeability. In addition, for the initial experiments aiming to monitor the peptide intracellular delivery, PS1-LNT was conjugated with the fluorescein isothiocyanate (FITC) (Fig. 2a).

As shown in Fig. 2, PS1-LNT-FITC at 5 μM can efficiently permeate the cells within 2-h incubation (Fig. 2b) without causing any toxicity (Fig. 2c). We also determined that PS1-LNT does not alter PS1 endoproteolysis, as demonstrated by the lack of significant changes in PS1 NTF and PS1 CTF generation (Fig. 2d).

**Fig. 3:**
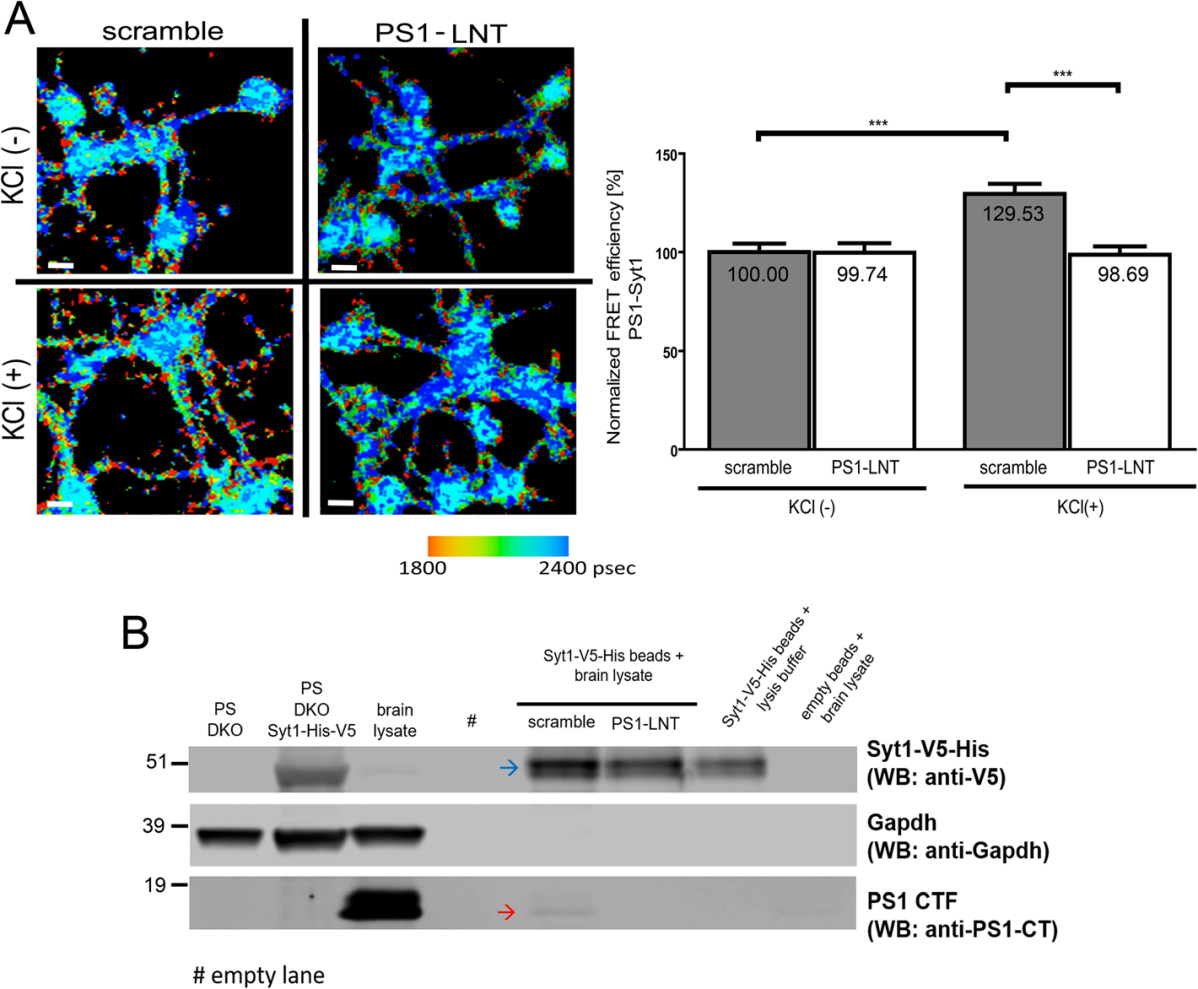
Inhibition of calcium-induced PS1-Syt1 interactions in primary neurons using PS1-LNT peptide. **a** The PS1-Syt1 proximity in neurons pre-treated with PS1-LNT or scramble peptide at the basal (KCl(-)) conditions or after 15-min depolarization with 50 mM KCl was determined by FLIM. The neurons were immunostained with anti-PS1 N-terminus (NT) and anti-Syt1 antibodies, followed by fluorescently conjugated secondary antibodies. The change in FRET efficiency was used to estimate relative change in the proximity between PS1 and Syt1. The pseudo-colored image presents the lifetime of the donor fluorophore in picoseconds. The orange-red pixels indicate shorter lifetimes reflecting closer proximity between the fluorescently labeled PS1 and Syt1; scale bar 10 μm. The graph shows normalized FRET efficiency between PS1 and Syt1. Note, PS1-LNT-mediated inhibition of the calcium-induced PS1-Syt1 interaction in neurons. The data are presented as mean ± SEM, *n* = 64 for scramble KCl (-), *n* = 70 for PS1-LNT KCl (-), *n* = 68 for scramble KCl (+) and *n* = 70 for PS1-LNT KCl (+), *n* = total number of neurons analyzed in 3 independent experiments. Statistical significance was determined using two-tailed unpaired Student’s t-test, ***p < 0.001. The fluorescence lifetimes and corresponding FRET efficiency values are shown in the Additional file 1. **b** In vitro IMAC analysis demonstrates successful inhibition of the PS1 binding to Syt1 by the PS1-LNT peptide. His-V5-Syt1 expressed in PS DKO cells was purified and immobilized on the beads using immobilized metal affinity chromatography, IMAC. The beads were incubated with mouse brain lysate in the presence of scramble or PS1-LNT peptide. The blue arrow indicates bands corresponding to the Syt1 immobilized on the beads. The red arrow shows the band corresponding to PS1 CTF bound to the immobilized Syt1 in the presence of scramble peptide only. Gapdh immunobloting was used as control. *n* = 3 independent experiments

Next, to assess if the PS1-LNT can inhibit the target interaction, we monitored proximity between endogenous PS1 and Syt1 in intact primary neurons. For this, the cells were pre-treated for 2 h with PS1-LNT or scramble peptide as a control, stimulated for 15 min with 50 mM KCl or H_2_O vehicle, immunostained with anti-PS1 NT and anti-Syt1 antibodies, and relative proximity between the fluorescently labeled proteins was determined by FLIM. PS1-LNT did not have any noticeable effect on the PS1-Syt1 interaction in the absence of KCl stimulation, due to relatively low intracellular calcium concentration and minimal spontaneous activity of the in vitro cultured primary neurons [41]. Thus, observed calcium-dependent PS1-Syt1 binding is relatively scarce at the basal condition, as visualized by the occasional red pixels on the pseudo-colored FLIM images (Fig. 3a). As expected, KCl-mediated synchronous calcium influx increased the relative FRET efficiency (PS1-Syt1 proximity) by 29.53 ± 5.15%, *p* < 0.0001 in scramble peptide pre-treated cells, similarly to that in non-pre-treated neurons [12] (Fig. 3a and Additional file 1). However, KCl failed to increase the E_FRET_ in the PS1-LNT pre-treated neurons (Fig. 3a and Additional file 1). These data demonstrate that the effect of the PS1-LNT peptide is the most pronounced in the KCl(+) condition, when it reduces the efficiency of the PS1-Syt1 binding.

The ability of the PS1-LNT peptide to impede calcium-dependent PS1-Syt1 interaction was further confirmed using a complementary approach: IMAC in vitro PS1-Syt1 binding assay. His-V5-Syt1, extracted from the PS DKO MEF cells transiently transfected with His-V5-Syt1, was immobilized on the beads, and the beads were incubated with mouse brain lysates, supplemented with 2 mM Ca^2+^, in the presence of scramble or PS1-LNT peptide. Binding of the endogenous PS1 to the immobilized His-V5-Syt1 was observed only in the presence of scramble peptide, and was blocked by the presence of PS1-LNT (Fig. 3b).

Together, these data confirm the ability of PS1-LNT peptide to inhibit calcium-triggered PS1-Syt1 interaction in primary neurons.

### PS1-Syt1 interaction modulates PS1 conformation and Aβ secretion

We have previously demonstrated that PS1/γ-secretase exists in a dynamic equilibrium of the distinct conformational states, so called “closed” and “open”, as determined by varied distances between the fluorescently labeled PS1 N-terminus and PS1-C-terminus/loop domains. The conformation of PS1 affects the position of the γ-secretase cleavage site on amyloid precursor protein (APP), and hence impacts generation of the different amyloid β (Aβ) species. Close PS1 domain arrangement, “closed” conformation, correlates with the increased Aβ42/40 ratio [12, 15, 21, 22].

**Fig. 4:**
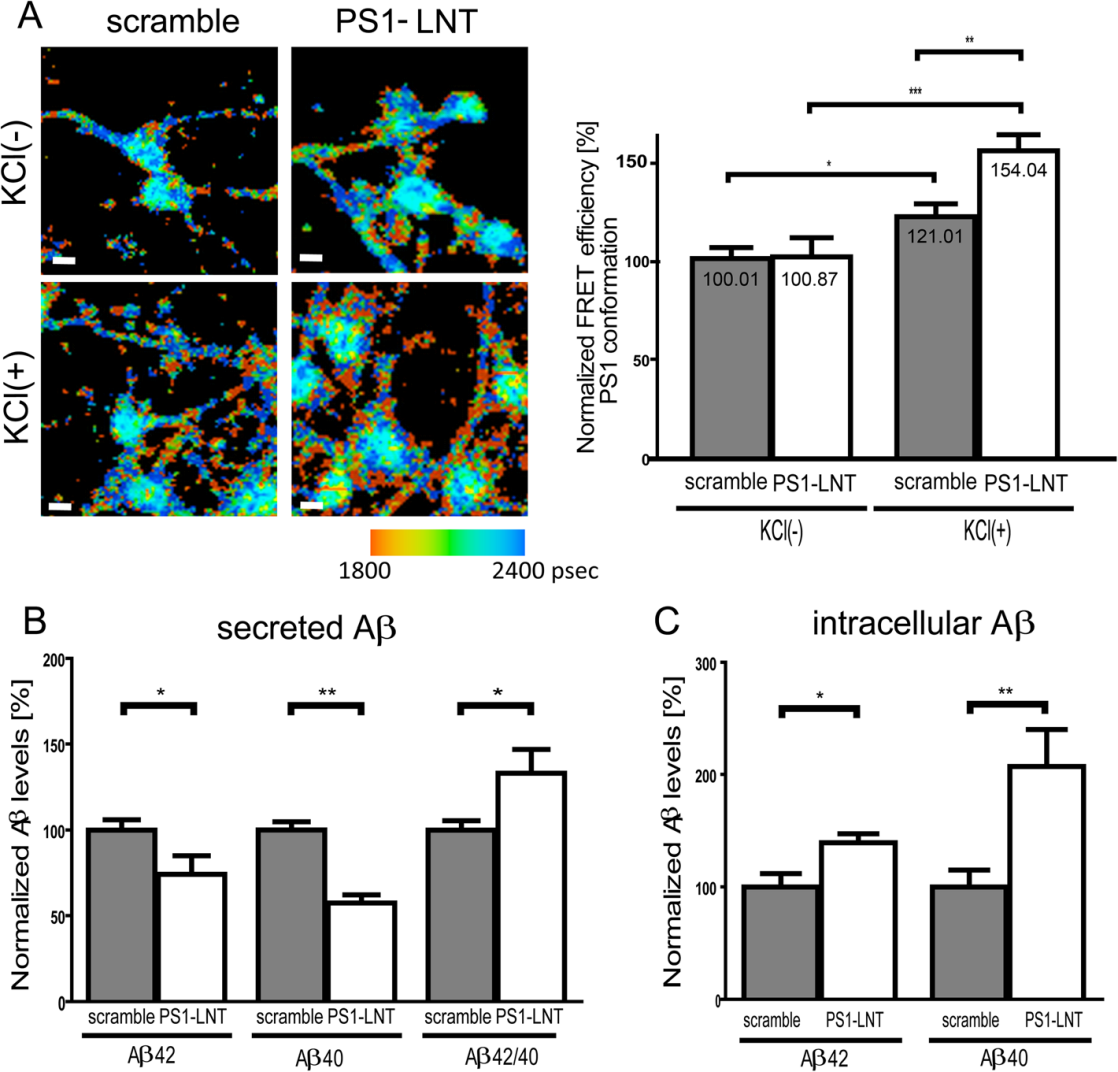
Activity-driven PS1-Syt1 interaction stabilizes open PS1 conformation and modulates Aβ production/secretion. **a** The conformation of endogenous PS1 in primary neurons pre-treated with PS1-LNT or scramble peptide at basal (KCl(-)) and high calcium (upon 15-min 50 mM KCl stimulation) conditions was determined by FLIM assay, monitoring proximity between PS1 N-terminus and PS1 loop domain. Cells were immunostained with anti-PS1 NT and anti-PS1 loop antibodies, followed by fluorescently conjugated secondary antibodies. The pseudo-colored images present the donor fluorophore lifetime in picoseconds. The orange-red pixels indicate PS1 in “closed” conformation; scale bar 10 μm. The adjacent graph presents normalized FRET efficiency between PS1-NT and PS1 cytosolic loop. The KCl stimulation shifts PS1 to “closed” conformation in both scramble and PS1-LNT pre-treated neurons but the shift is greater in the PS1-LNT conditions. The data are presented as mean ± SEM, *n* = 64 for scramble (KCl(-)), *n* = 57 for PS1-LNT (KCl(-)), *n* = 59 for scramble (KCl(+)) and *n* = 65 for PS1-LNT (KCl(+)), *n* = number of neurons analyzed in 3 independent experiments. Statistical significance was determined using two-tailed unpaired Student’s t-test, **p < 0.01. The fluorescence lifetimes and corresponding FRET efficiency values are shown in the Additional file 1. **b** The graph presents relative Aβ40, Aβ42 levels and Aβ42/40 ratio in conditioned medium collected from the neurons pre-treated with PS1-LNT and depolarized with KCl to induce calcium influx. The values, determined by ELISA, were normalized to the scramble treated cells. The data are presented as mean ± SEM, *n* = 19-26. Statistical significance was determined using two-tailed unpaired Student’s t-test, *p < 0.05, **p < 0.01. **c** The graph presents relative Aβ40 and Aβ42 levels in cell lysates prepared from the neurons used in (**b**). The values were normalized to the scramble treated cells. The data are presented as mean ± SEM, *n* = 7-12. Statistical significance was determined using two-tailed unpaired Student’s t-test, *p < 0.05, **p < 0.01

To determine the role of the PS1-Syt1 interaction in the modulation of PS1 conformation and Aβ production, PS1 conformation was assayed in non-stimulated and KCl-stimulated neurons, pre-treated with PS1-LNT or scramble peptide, using antibody-based FLIM. The 15-min KCl application triggered an increase in the FRET efficiency, indicative of the shorter distance between the fluorophores labeling PS1 NT and loop domains, in both scramble and PS1-LNT pre-treated neurons, consistent with the previous data for non-pre-treated cells [12]. Interestingly, this increase was significantly higher in the PS1-LNT pre-treated neurons, where binding to the Syt1 was blocked, compared to the scramble/control cells (Fig. 4a and Additional file 1). This suggests that Syt1 via binding to PS1 lessens the strong, “pathogenic” closure of the PS1 conformation caused by high calcium.

To test whether inhibition of the PS1-Syt1 interaction would affect Aβ production/secretion, the Aβ42 and Aβ40 levels in the conditioned medium of the PS1-LNT and control pre-treated neurons were measured. We found that inhibition of the interaction between PS1 and Syt1 results in a significant increase in the Aβ42/40 ratio, although individual levels of the Aβ40 and Aβ42 were reduced (Fig. 4b).

The reduced Aβ levels in the conditioned medium could be a consequence of the changed APP expression and/or processing, or impaired Aβ secretion. To distinguish between these possibilities, we evaluated the levels of APP full length (APP-FL) and APP C-terminal fragments (APP-CTFs) in KCl-stimulated scramble and PS1-LNT pre-treated neurons. No accumulation of the APP-CTFs was observed upon the inhibition of the PS1-Syt1 binding, suggestive of the lack of alterations in the overall PS1/γ-secretase activity (Additional file 2).

To explore if the observed decrease in the Aβ amount in the conditioned medium is indeed a consequence of the impaired Aβ secretion, we measured intracellular Aβ42 and Aβ40 levels in the control or PS1-LNT pre-treated, KCl-stimulated neurons (Fig. 4c). An increased amount of the intracellular Aβ42 and Aβ40 was detected in the cells pre-treated with PS1-LNT compared to the control, suggesting that inhibition of the PS1-Syt1 binding may result in aberrant exocytosis. These results were further supported by double-immunostaining of neurons with the anti-β-amyloid antibody 4G8, recognizing both Aβ and APP, and anti-microtubule associated protein 2 (MAP2) antibody as an internal control. Since we did not see difference in the APP-FL and APP-CTFs between the scramble and PS1-LNT pre-treated cells, increased 4G8 fluorescence intensity in PS1-LNT pre-treated cells (Additional file 3) suggests accumulation of the intracellular Aβ.

**Fig. 5:**
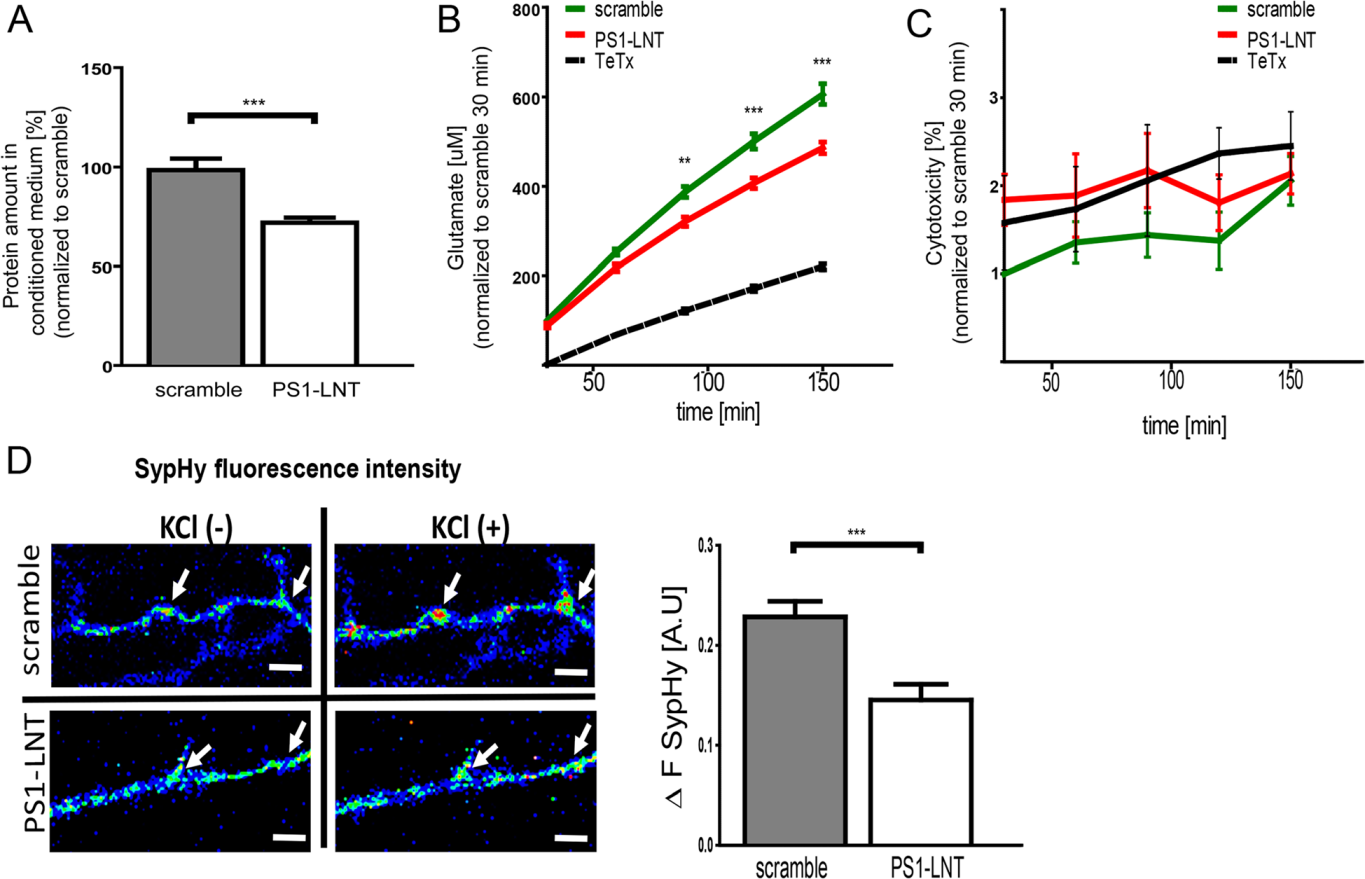
PS1-Syt1 interaction modulates exocytosis/neurotransmitter release. **a** The graph presents the quantification of the total protein amount in the conditioned medium collected from neurons plated at 1.5x10^6^ cells/well, pre-treated for 2 h with scramble and PS1-LNT peptides, and stimulated for 15 min with 50 mM KCl. The protein secreted by the PS1-LNT pre-treated neurons is shown as a percent of the protein secreted by the scramble peptide pre-treated cells; mean ± SEM, *n* = 12. Statistical significance was determined using two-tailed unpaired Student’s t-test, ***p < 0.001. **b** The graph presents relative amounts of glutamate in the conditioned medium collected from neurons pre-treated for 2 h with PS1-LNT, scramble peptide or exocytosis inhibitor, tetanus toxin (TeTx), and depolarized using 50 mM KCl in the presence of the DL-TBOA, an inhibitor of the glutamate transporters EAAT1 and EAAT2. The data are presented as mean ± SEM, *n* = 31 for scramble and PS1-LNT, *n* = 6 for TeTx. Statistical significance was determined using two-way ANOVA followed by Bonferroni’s post-test, **p < 0.01, *** < 0.001. **c** The graph presents relative level of lactate dehydrogenase activity, indicative of the potential cytotoxicity of the applied treatment, in primary neurons pre-treated with PS1-LNT or scramble peptide for 2 h and depolarized using 50 mM KCl in the presence of the DL-TBOA for the duration of the assay. The data are presented as mean ± SEM, *n* = 7. Statistical significance was determined using two-way ANOVA followed by Bonferroni’s post-test. No significant toxicity of the applied treatments was observed. **d**, The pseudocolor-coded images present synaptophluorin (SypHy) fluorescence at the synaptic puncta in neurons pre-treated for 2 h with PS1-LNT or scramble peptide. The red spots indicate high fluorescence intensity corresponding to the high level of exocytosis observed immediately after the 50 mM KCl stimulation; scale bar 5 μm. The adjacent bar graph demonstrates the quantification of the increase in SypHy fluorescence at the synaptic ROIs in response to the depolarizing stimulus in the scramble and PS1-LNT pre-treated neurons. The data are presented as mean ± SEM, *n* = 263 for scramble and *n* = 255 for PS1-LNT, *n* = number of synaptic puncta analyzed in 4 independent experiments. Statistical significance was determined using Mann-Whitney’s *U*-test, ***p < 0.01

Next, we determined whether PS1-LNT impaired exocytosis is specific for Aβ or represents more general phenomena. Multiple proteins are released from neurons in an activity-dependent exocytosis controlled by synaptic proteins [42–44]. Thus, we used an accurate and ultrasensitive NanoOrange assay to monitor changes in the total protein concentration in the conditioned medium of neurons pre-treated for 2 h with PS1-LNT or scramble peptide, and stimulated with 50 mM KCl for 15 min. Indeed, the amount of total protein detected in the conditioned medium of PS1-LNT pre-treated neurons was significantly reduced, compared to that of scramble pre-treated cells (Fig. 5a). This further supports overall impairment of exocytosis/secretion after the inhibition of the Syt1-PS1 binding.

### PS1-Syt1 interaction stimulates exocytosis and neurotransmitter release in primary neurons

Syt1 is primarily expressed at the synapse and calcium influx has been shown to predominantly trigger PS1-Syt1 interaction at the synaptic loci [12]. This rises a possibility that PS1-Syt1 binding may modulate synaptic vesicle exocytosis and neurotransmitter release. Therefore, to examine whether the PS1-Syt1 interaction indeed modulates synaptic exocytosis we monitored the level of secreted glutamate over the 150-min time course in the conditioned medium of KCl-stimulated neurons pre-treated for 2 h with 5 μM of either PS1-LNT or scramble peptides. A blocker of the excitatory amino acid transporters 1 and 2 (EAAT1, EAAT2), DL-threo-beta-benzyloxyaspartate (DL-TBOA), was used to inhibit glutamate re-uptake. The inhibition of the PS1-Syt1 binding by PS1-LNT resulted in a significant reduction of the glutamate level in the conditioned medium (Fig. 5b), suggesting importance of the PS1-Syt1 interaction for glutamate secretion/exocytosis. Overnight pre-treatment of the neurons with tetanus toxin (TeTx), a commonly used potent inhibitor of the exocytosis [45], markedly diminished glutamate release, and served as an assay control. Importantly, the described reductions in glutamate release in the PS1-LNT and TeTx pre-treated neurons were not due to increased cytotoxicity (Fig. 5c).

The role of PS1-Syt1 interactions in synaptic vesicle exocytosis was further assessed using an alternative approach, monitoring fluorescence intensity of the pH-sensitive reporter of the synaptic vesicle exocytosis, synaptophluorin (SypHy) [33]. The SypHy was expressed in 13 DIV neurons, and changes in its fluorescence intensity pre- and post-KCl stimulation were monitored in real time in live cells pre-incubated with PS1-LNT or scramble peptide. No marked differences in the SypHy expression were observed between PS1-LNT and scramble peptide treated neurons at the baseline conditions (Fig. 5d, KCl(-)). As expected, the fluorescence intensity increased immediately after the KCl application, indicative of synaptic vesicle exocytosis, but the increase was less prominent in PS1-LNT than in scramble treated neurons (Fig. 5d, KCl(+)).

Collectively, the decreased level of Aβ and glutamate in the conditioned medium, and reduced rise in the SypHy fluorescence in KCl-stimulated neurons after inhibition of the PS1-Syt1 interactions strongly suggest that the PS1-Syt1 binding may help upkeep exocytosis in primary neurons.

### PS1-Syt1 binding influences trafficking of synaptic vesicles

**Fig. 6:**
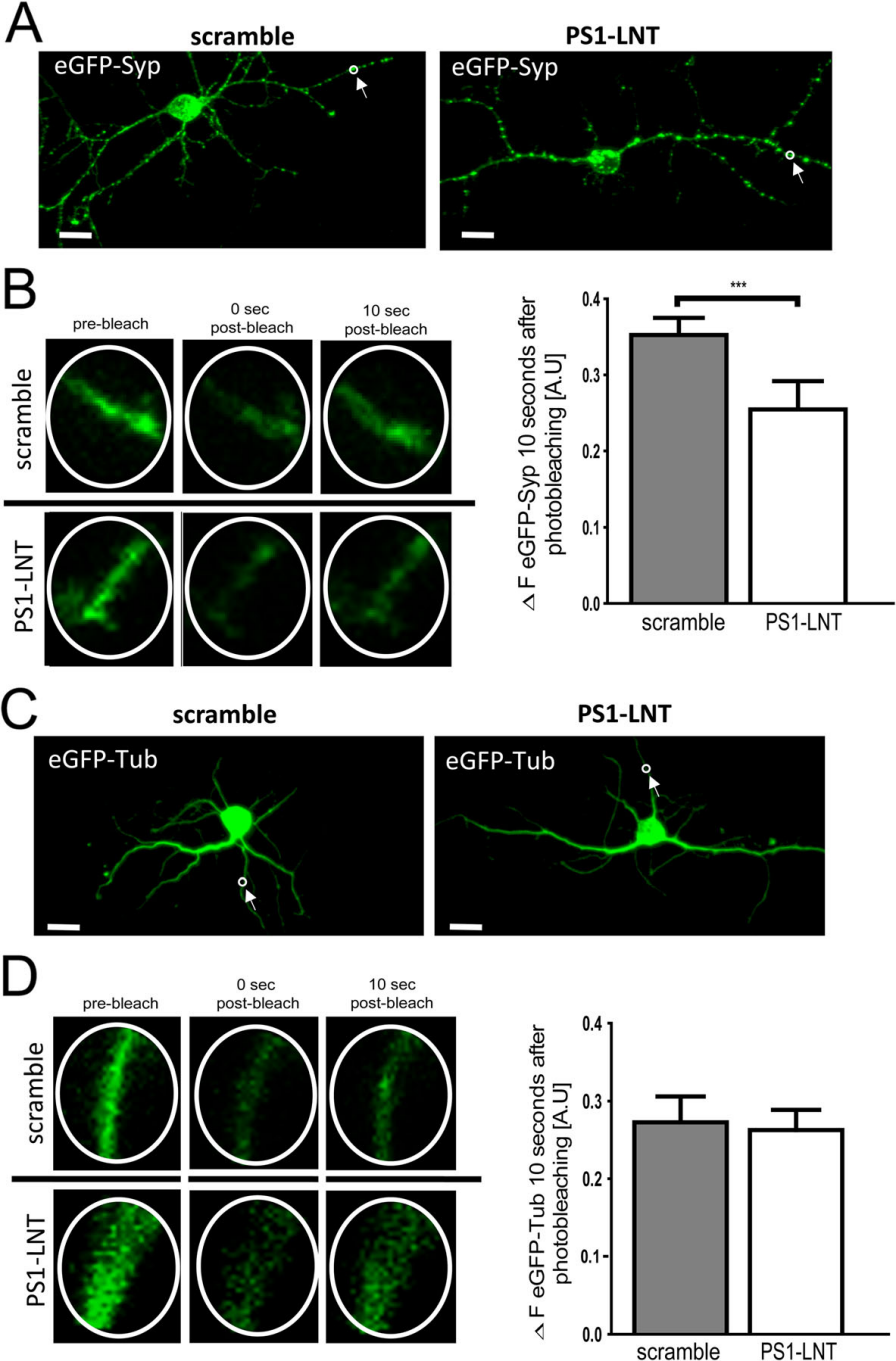
PS1-Syt1 interaction modulates synaptic vesicle trafficking. **a** Fluorescent images show distribution of the eGFP-synaptophysin (eGFP-Syp) in primary neurons pre-treated for 2 h with scramble and PS1-LNT peptides. Note increased fluorescence intensity in synaptic puncta along the processes. The arrows point to the example region of interests (ROIs) used for the photobleaching experiments; scale bar 15 μm. **b** Fluorescence recovery after photobleaching (FRAP) experiments demonstrate impaired motion of synaptic vesicle proteins in the PS1-LNT pre-treated neurons. The fluorescent images present 5 μm-diameter ROIs recorded in eGFP-Syp expressing neurons pre-treated for 2 h with scramble or PS1-LNT peptides, and stimulated for with 50 mM KCl. Three ROIs are analyzed for each condition: pre-bleach, immediately after bleach and 10 s after bleach. The adjacent bar graph demonstrates the quantification of the eGFP-Syp fluorescence recovered within 10 s post-bleach. The data are presented as mean + SEM, *n* = 36. Statistical significance was determined using unpaired Student’s t-test, ***p < 0.001. **c** Fluorescent images present distribution of the eGFP-tubulin (eGFP-Tub) in primary neurons pre-treated with scramble and PS1-LNT peptides. The arrows point to the ROIs used for the photobleaching experiments; scale bar 15 μm. **d** Control FRAP experiments demonstrate no alterations in eGFP-tubulin fluorescence recovery after photobleaching in neurons pre-treated for 2 h with PS1-LNT or scramble peptides, and stimulated with 50 mM KCl for 15 min. The fluorescent images present 5 μm-diameter ROIs recorded in eGFP-Tub expressing neurons pre-treated with scramble or PS1-LNT peptides. The data are presented as mean + SEM, *n* = 19. Statistical significance was determined using unpaired Student’s t-test

To further address the role of PS1-Syt1 interaction in synaptic physiology, the mobility of synaptic vesicles along the processes was investigated using fluorescence recovery after photobleaching (FRAP) of the eGFP-synaptophysin (eGFP-Syp). eGFP-Syp has been previously reported to be predominantly expressed in axons and to co-localize with the other synaptic vesicle markers [46, 47]. The neurons expressing eGFP-Syp were pre-treated with 5 μM scramble or PS1-LNT peptides for 2 h and stimulated for 15 min with 50 mM KCl to promote PS1-Syt1 interaction. The inhibition of the PS1-Syt1 binding by PS1-LNT led to decreased recovery rate of the eGFP-Syp fluorescence (Fig. 6a,b), suggestive of compromised synaptic vesicle movement in PS1-LNT vs. scramble peptide pre-treated cells. Of note, FRAP of the eGFP-tubulin, which served as a negative control, was not altered by the PS1-LNT pre-treatment (Fig. 6c,d).

### Inhibition of PS1-Syt1 interaction leads to alterations in the synaptic vesicle morphology and distribution

**Fig. 7:**
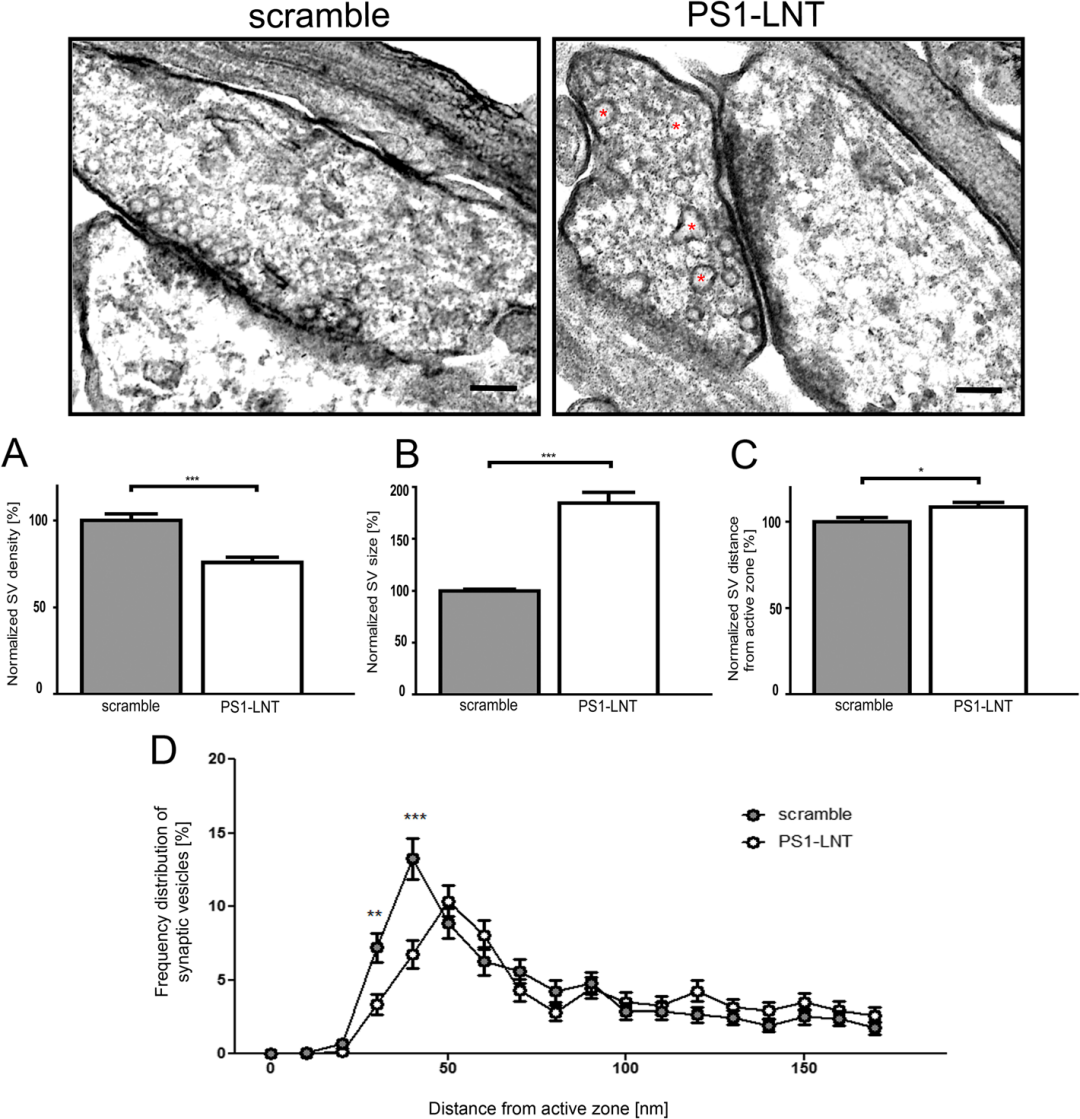
Inhibition of PS1-Syt1 binding leads to the alterations in synaptic vesicle morphology and distribution. Representative electron micrographs show synaptic vesicles within the synapses of the primary neurons pre-treated for 2 h with scramble or PS1-LNT peptides, and stimulated for 15 min with 50 mM KCl. Enlarged synaptic vesicles in the PS1-LNT treated synapses are shown by red asterisks. Scale bar 100 nm. The graphs show the quantification of: **a** synaptic vesicle size; **b** density; and **c** distance from the active zone. The data are normalized to scramble-treated cells and presented as mean ± SEM, *n* = 100, *n* = total number of synapses analyzed in three independent experiments. Graph (**d**) presents the frequency distribution of the synaptic vesicles within individual synapses as a function of the distance from the active zone. Statistical significance was determined using two-tailed unpaired Student’s t-test (**a**, **b**, **c**) or two-way ANOVA followed by Bonferroni’s post-test (**d**), *p < 0.05, **p < 0.01, ***p < 0.001

To determine if PS1-LNT interference with the PS1-Syt1 binding may lead to ultrastructural alterations at the synapse, we performed electron microscopy (EM) analysis of the synaptic vesicle size, density and distance from the active zone in primary neurons pre-treated for 2 h with scramble or PS1-LNT peptide, and stimulated for 15 min with 50 mM KCl (Fig. 7). A population of significantly larger synaptic vesicles and reduced overall synaptic vesicle density were detected in synaptic terminals of the PS1-LNT pre-treated neurons compared to the control. Moreover, an increase in the distance of the synaptic vesicle to the active zone was observed upon the inhibition of the PS1-Syt1 binding.

### Inhibition of the PS1-Syt1 interaction leads to the loss of dendritic spines

**Fig. 8:**
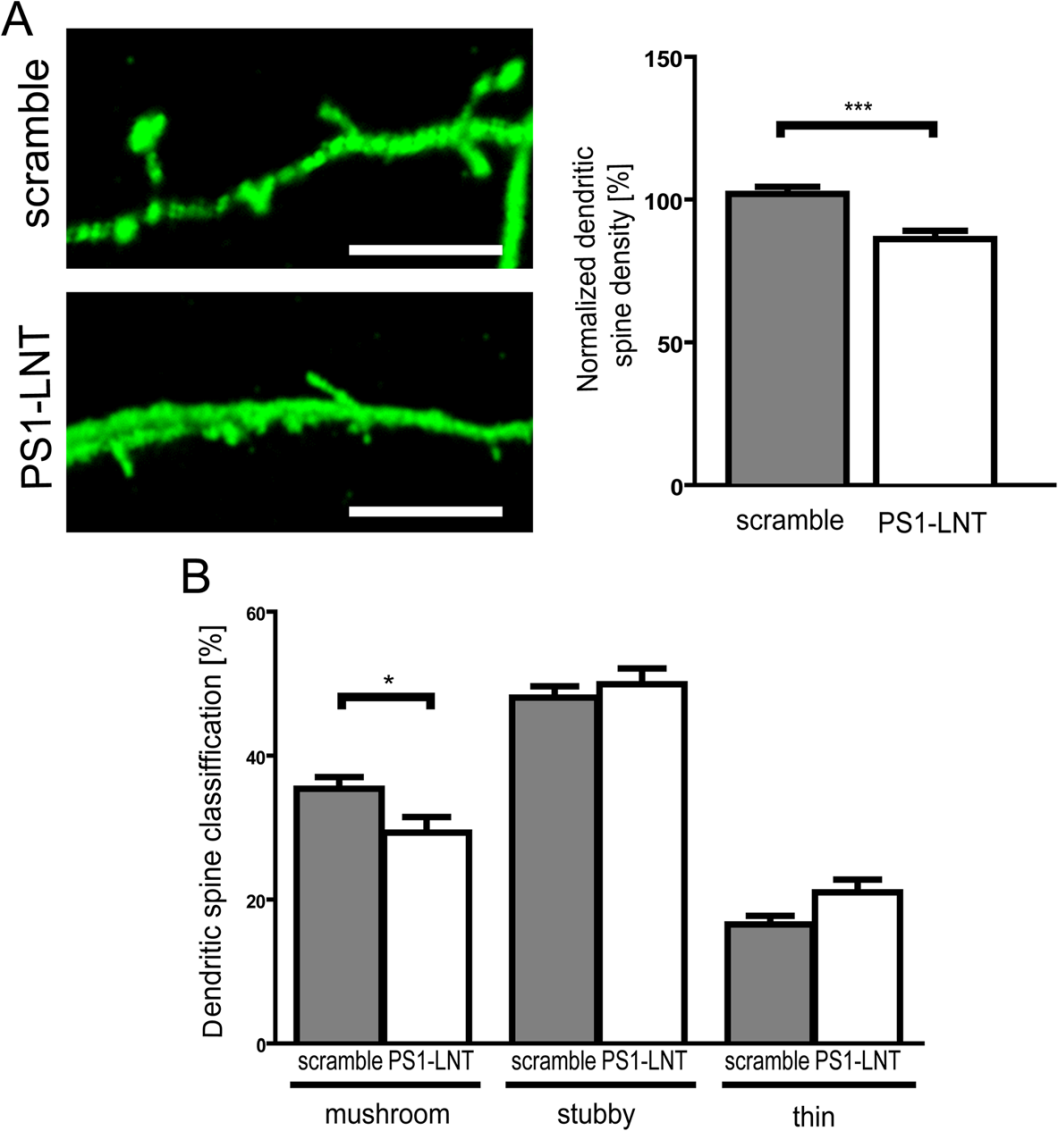
Inhibition of PS1-Syt1 interaction alters the number and the morphology of dendritic spines. **a** Representative images of the dendritic spines in GFP overexpressing neurons, pre-treated for 2 h with scramble or PS1-LNT peptides, and stimulated for 15 min with 50 mM KCl. Scale bar 5 μm. The adjacent bar graph shows the quantification of the dendritic spine density. The values are normalized to the scramble treated cells. The data are presented as mean ± SEM, *n* = 30, *n* = number of neurites analyzed per condition in 8 independent experiments. Statistical significance was determined using two-tailed unpaired Student’s t-test, ***p < 0.01. **b** The bar graph presents quantitative analysis of the relative contribution of the mushroom, stubby and thin dendritic spines to the total spine number in neurons shown in (**a**). The data are presented as mean ± SEM, *n* = 50 for scramble and *n* = 43 for PS1-LNT, *n* = number of images acquired per condition in 8 independent experiments. Statistical significance was determined using two-way ANOVA followed by a Bonferroni’s post-test, *p < 0.05

In order to investigate the pathophysiological relevance of the observed abnormalities in the exocytosis, synaptic vesicle trafficking and ultrastructural impairments, we analyzed the number and the morphology of the dendritic spines in KCl-stimulated scramble or PS1-LNT peptide pre-treated neurons. The inhibition of the PS1-Syt1 interaction resulted in the reduction of the total number of dendritic spines (Fig. 8). In addition, when the dendritic spines were classified according to their shape into mushroom-shaped, stubby or thin, the decrease in the fraction of mushroom spines was apparent in the PS1-LNT pre-treated neurons. This further reaffirms the importance of the PS1-Syt1 binding for the physiology of the synapse.

### Syt1 level and PS1-Syt1 proximity are decreased in Alzheimer’s disease brains

**Fig. 9:**
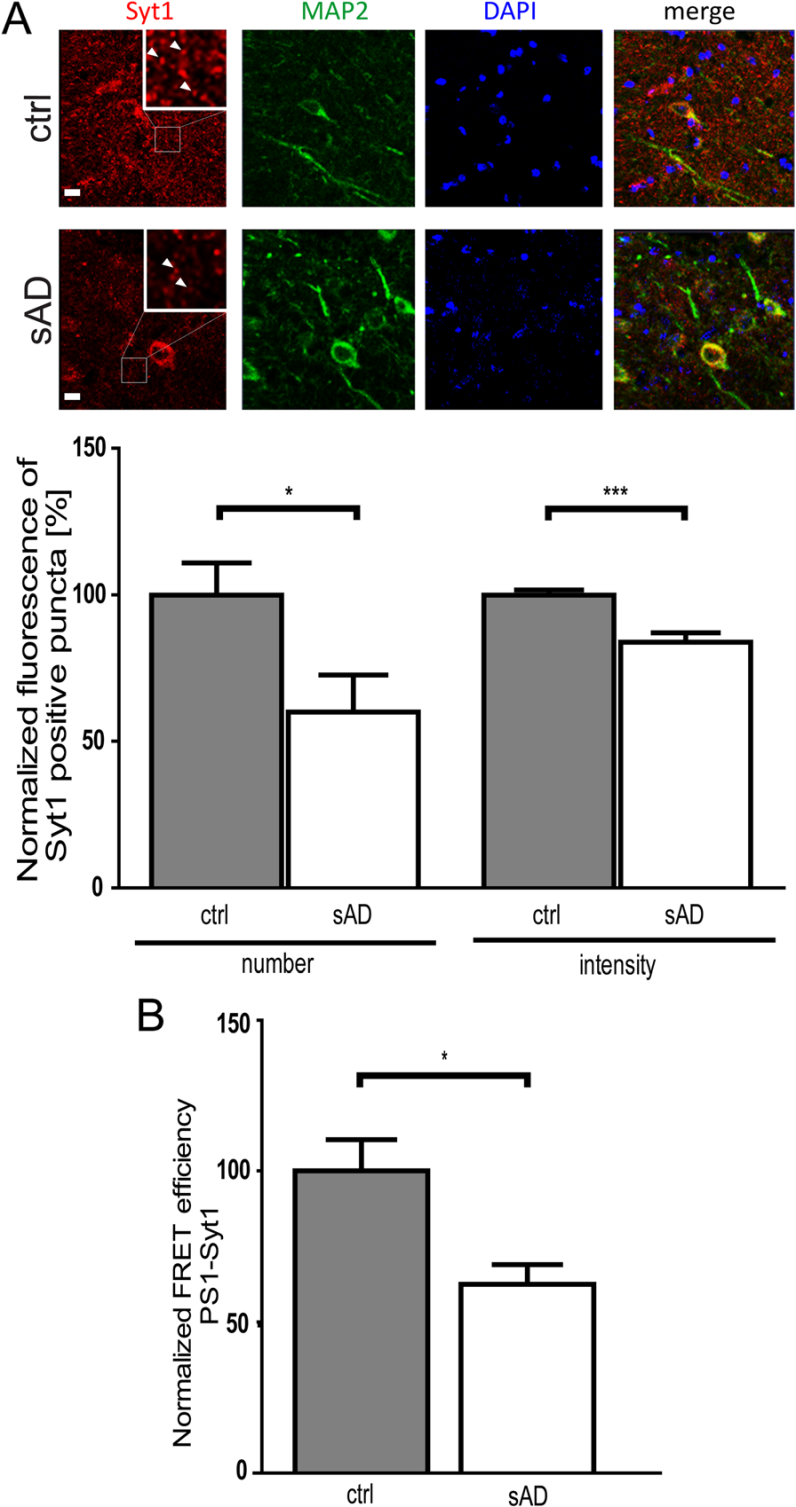
Reduced Syt1 level and PS1-Syt1 interactions in human sporadic AD brains. **a** Representative images of the Syt1 immunoreactivity in the medial temporal cortex of control and Braak V-VI stage sporadic AD brains; scale bar 10 μm. The inserts present magnified areas from the images and demonstrate the individual synaptic puncta (arrows). The bar graph presents quantification of the number and intensity of the Syt1-positive puncta in control vs. sAD patients. The data are presented as mean ± SEM, *n* = 4, 4–5 fields analyzed for each case. Statistical significance was determined using two-tailed unpaired Student’s t-test. *p < 0.05, **p < 0.01. There is a significant reduction in the Syt1 immunofluorescence in sAD brains. **b** FLIM analysis of PS1-Syt1 interactions in medial temporal cortex of control vs. sAD presents reduced PS1-Syt1 binding in sAD. The tissue was immunostained with anti-PS1 NT (APS11) and anti-Syt1 antibodies, and relative proximity between the fluorescently labeled proteins was determined by FLIM. There is significant reduction in the FRET efficiency between PS1 and Syt1, indicative of a greater distance between the molecules, in sAD brains. The data are presented as mean ± SEM, *n* = 4; 20–25 neurons analyzed for each case. Statistical significance was determined using Mann-Whitney’s *U*-test. *p < 0.05. The fluorescence lifetimes and corresponding FRET efficiency values are shown in the Additional file 1

Synaptic defects, impaired exocytosis, and pathogenic change in the PS1 conformation were reported to occur in AD [38, 48–51]. Since our data show that these were affected by the inhibition of the PS1-Syt1 interaction, we analyzed Syt1 protein expression and the relative proximity between PS1 and Syt1 in medial temporal cortex from sporadic AD (sAD) cases with neuropathologically confirmed Braak stage V-VI, and in age- and post mortem interval (PMI)-matched control subjects. The number and fluorescence intensity of the Syt1-positive puncta were significantly decreased in sAD brains (Fig. 9a).

To determine if the proximity between the remaining Syt1 and PS1 is also altered in sAD, we employed an antibody-based FLIM assay. FLIM approach determines the fluorophore lifetime, which is an intrinsic biophysical property of a fluorophore, and hence does not depend on the absolute amounts of the donor and the acceptor in the sample [36, 52, 53]. Therefore, despite significant reduction in the absolute levels of synaptic proteins in AD brain, FLIM is uniquely suited to report the relative distance between the remaining Syt1 and PS1. Longer lifetimes of the donor fluorophore, indicative of the lower FRET efficiency, stemming from reduced proximity between the fluorescently labeled PS1 and Syt1, were recorded in sAD compared to control cases (Fig. 9b and Additional file 1). This indicates that in addition to decreased Syt1 level, the interaction between Syt1 and PS1/γ-secretase in sAD brains is also significantly reduced.

### Syt1 overexpression in vivo in mouse brain promotes protective “open” PS1 conformation

**Fig. 10:**
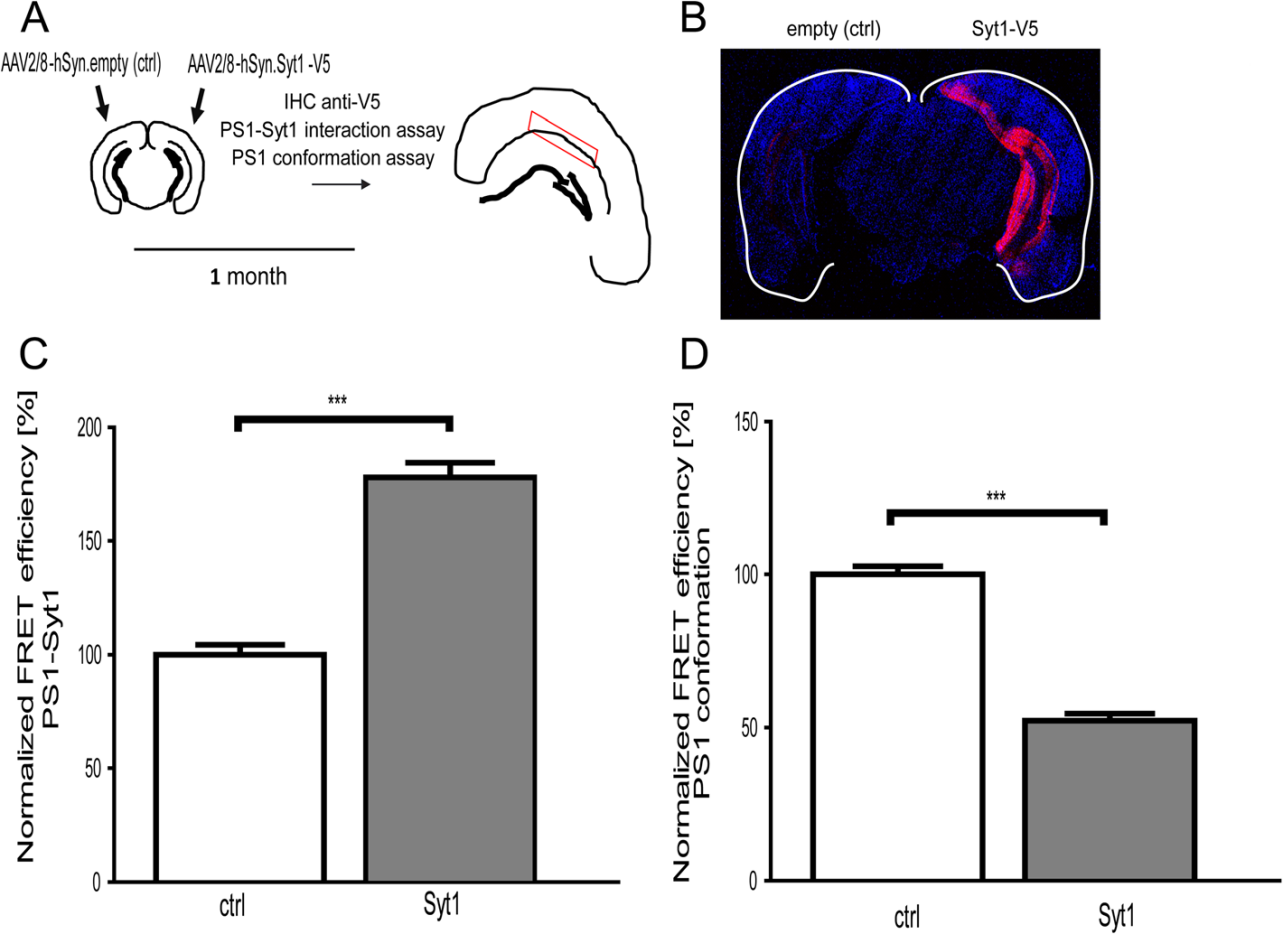
Syt1 overexpression in vivo increases PS1-Syt1 interactions and promotes open PS1 conformation in mouse brain. **a** Schematic representation of the experimental design. Bilateral injection of AAV2/8-hSyn1. Syt1-V5 or empty plasmid to the contralateral hemisphere was performed. One month after the injections the brains were dissected and subjected to immunofluorescence analysis. **b** The fluorescent images demonstrate successful expression of Syt1-V5 in mouse brain one month after the AAV2/8-hSyn1. Syt1-V5 injection. The tissue was immunostained with anti-V5 antibody and corresponding Cy3-conjugated secondary antibody. **c**, **d** The bar graphs present FLIM assays of the PS1-Syt1 interactions and PS1 conformation in mouse hippocampi injected with AAV2/8-hSyn1. Syt1-V5 or empty vector as a control. The tissue was immunostained with anti-PS1 NT and anti-Syt1 for the PS1-Syt1 interaction assay, or anti-PS1 NT and anti-PS1 loop antibodies for the PS1 conformation assay, followed by respective fluorescently labeled secondary antibodies. There is a significant increase in the PS1-Syt1 binding and a shift towards protective “open” conformation of PS1 in the hemispheres transduced with AAV2/8-hSyn1. Syt1-V5. The data are presented as mean ± SEM, *n* = 721-723 neurons for PS1-Syt1 interaction and *n* = 573-586 for PS1 conformation were analyzed in 5 mice. Statistical significance was determined using Mann-Whitney’s *U-*test, ***p < 0.001. The fluorescence lifetimes and corresponding FRET efficiency values are shown in the Additional file 1

To investigate whether overexpression of Syt1 in vivo in mouse hippocampi would promote the PS1-Syt1 interaction, and thus support the protective “open” PS1 conformation, we employed adeno-associated virus (AAV2/8)-mediated delivery of the Syt1-V5 or empty vector control plasmids into the hippocampi of wild type C57BL/6 mice. To minimize variability between the animals Syt1-V5 was expressed in one hemisphere and an empty vector was delivered to the contralateral side (Fig. 10a). One month post-injection the brains were dissected and the Syt1-V5 expression in the targeted hemisphere was confirmed by immunostaining with anti-V5 antibody (Fig. 10b). We found that increased Syt1 expression promoted the interaction between PS1 and Syt1, as determined by antibody-based FLIM assay (Fig. 10c and Additional file 1).

Next, we analyzed if increased Syt1 expression and its interaction with PS1 would promote “open” PS1 conformation in vivo. Significantly decreased FRET efficiency, indicative of a longer distance between fluorescently labeled PS1 N-terminus and large cytosolic loop domain, was observed in the hemisphere injected with the AAV2/8-Syt1-V5 compared to the contralateral control hemisphere (Fig. 10d and Additional file 1). This reaffirms that Syt1 expression in vivo helps sustain protective “open” PS1 conformation and might be worth exploring therapeutically.

## Discussion

By using targeted inhibition of the presenilin 1 (PS1) and synaptotagmin 1 (Syt1) binding, without affecting the proteins’ expression levels, we provide evidence of the reciprocal relationship between PS1 and synaptic vesicle protein Syt1, by showing that PS1 and Syt1 modulate each other’s functions. Furthermore, we found that proximity between the Syt1 and PS1 is significantly reduced in sporadic Alzheimer’s disease (sAD) brain, indicating physiological relevance of the PS1-Syt1 interaction, and suggesting that disrupted interaction may contribute to the synaptic pathology that starts early in the disease.

A link between abnormal neuronal/synaptic activity and Aβ accumulation has been reported [54–56] although the precise molecular mechanisms regulating synaptic Aβ generation and release remain elusive. We found that blocking KCl-triggered PS1-Syt1 interaction affected both the type of Aβ species generated and their release, without affecting overall γ-secretase activity or APP processing. First, targeted, peptide-based inhibition of the PS1-Syt1 binding exacerbated KCl-induced shift of PS1 towards the pathogenic “closed” state, and caused concurrent increase in the Aβ42/40 ratio. This suggests that Syt1 may serve as a protective factor stabilizing “open” PS1/γ-secretase conformation at the synapse at high calcium conditions. Second, we found that inhibition of the interaction between PS1 and Syt1 resulted in the reduced overall protein secretion and neurotransmitter release, and increased accumulation of the intracellular Aβ, indicating that PS1 may be involved in Syt1-mediated vesicle trafficking and exocytosis in primary neurons.

The potential role of PS1 at the synapse beyond γ-secretase activity remains poorly understood. The compromised neurotransmission has been observed in conditional pre- but not post-synaptic PS double knock-out neurons (PS cDKO), proposing a role of PS1 in the modulation of neurotransmitter release [23]. Since PS1 interacts with the ryanodine receptor (RyR) [57], and PS cDKO mice present reduced RyR level [58], the impaired neurotransmission in PS cDKO neurons has been attributed to the aberrant calcium release through RyR [23]. On the other hand, γ-secrease activity-dependent PS1 involvement in regulation of the spontaneous neurotransmitter release was also shown in PS1 knock-out neurons [24]. However, given the pleiotropic effect of protein knock-out and considering multiple functions of presenilins, as shown by the global PS1-dependent transcriptome changes [59], PS1 knock-out approach does not provide evidence of the direct involvement of PS1 in synaptic vesicle exocytosis/neurotransmission.

Our discovery of the calcium-regulated PS1 interaction with the synaptic vesicle release protein, Syt1, further alluded to the possibility that PS1 may play an important regulatory role at the synapse. Since Syt1 is directly involved in calcium-triggered exocytosis that depends on its precise, sequential interactions with the SNARE complex proteins, allowing vesicle fusion to occur and exocytosis to proceed [27–29, 60, 61], we hypothesized that PS1 binding to Syt1 may modulate this process. Consistent with this idea, our findings demonstrate that targeted inhibition of the PS1 interaction with one of the members of synaptic vesicle release machinery diminishes the activity driven exocytosis/glutamate release. Moreover, reduced recovery rate of the eGFP-synaptophysin fluorescence after photobleaching suggests compromised synaptic vesicle trafficking along the neuronal processes when the PS1-Syt1 interaction is impeded. These impairments may manifest with an altered distribution of the vesicles at the synapses. Indeed, electron microscopy analysis revealed enlarged, less densely packed synaptic vesicles that are positioned farther from the active zone in KCl-stimulated neurons after the inhibition of the PS1-Syt1 binding. These data suggest that PS1-LNT interference with the PS1-Syt1 interaction may lead to alterations in synaptic vesicle morphology/distribution at the synapse and compromise exocytosis. It is plausible that PS1 via the direct interaction with Syt1 regulates its structural flexibility and/or presentation of specific motifs on Syt1 necessary for the interaction with other synaptic partners crucial for synaptic vesicle exocytosis [62–65]. On the other hand, accumulation of the enlarged vesicles might indicate that the PS1-Syt1 binding regulates the Syt1-dependent mechanisms controlling synaptic vesicle size [66]. Of note, analogous structural abnormalities in the synaptic vesicles have been observed in Syt1 knock-out synapses [67, 68], but not in the Syt1 calcium-binding motif mutants [67], indicating that this is not a Syt1 calcium-binding defect.

The impairments in the exocytosis and neurotransmitter release at the pre-synapse due to the inhibition of the PS1-Syt1 interaction lead to the concomitant reduction in the number of dendritic spines, and mushroom spines in particular. This finding is consistent with the previous observations of decreased spine density in vivo following the expression of potent exocytosis inhibitors [69] and in AD models, as reviewed in [70, 71]. Together, with our findings of the detrimental effect of PS1-LNT blocking peptide treatment on PS1 conformation, Aβ42/40 ratio and intracellular Aβ accumulation, this further supports the beneficial role of the PS1-Syt1 interaction for synaptic wellbeing.

The described pathophysiological changes were detected in vitro upon profound but relatively short-lasting inhibition of the PS1-Syt1 binding. It is plausible that chronic, lasting over decades inhibition of the PS1-Syt1 interaction, even if more subtle, would lead to the neuronal circuitry dysfunction, and ultimately to AD. In accordance, several studies have reported changes in the synaptic physiology and exocytosis, such as alterations in RCAN1-mediated modulation of exocytosis in AD [49, 51] or exocytosis impairments in the cells from APP/PS1 mouse model of AD [50].

Synaptic dysfunction/loss is one of the major neuropathological feature of the disease, and the strongest correlate of the disease progression [1–3, 7, 48, 72]. The described protective Syt1 function might be compromised in AD brains, since, in agreement with other reports, we found reduced levels of Syt1 in sAD brains (this study and [7, 73]). Intriguingly, reduced Syt1 level in the brain is followed by its concomitant decrease in the cerebrospinal fluid and plasma neuronal-derived exosomes, raising the prospect for Syt1 being used as a potential biomarker for synaptic AD pathology [74, 75].

Furthermore, by applying fluorescence lifetime imaging microscopy, we found that the proximity between the remaining Syt1 and PS1 is also reduced in the disease. Of note, we have observed that the PS1-Syt1 interaction in mouse brain increases during normal aging (unpublished data). Therefore, it is possible that the PS1-Syt1 proximity is also higher in normal aged human brain (perhaps as a compensatory mechanism) but not in the sporadic AD brain. There is an array of factors that could potentially affect the baseline PS1-Syt1 interaction in aged brain. Despite the lack of PS1 mutations, the wild type PS1 in sAD brain presents structural alteration, i.e. pathogenic “close” conformation that is linked to elevated Aβ42/40 ratio [38, 76, 77]. Therefore, it is possible that conformational changes in PS1 might result in reduced binding to Syt1. It is also possible that reduction in the PS1-Syt1 binding can occur early, before the onset of symptoms, since ~30% decrease in the PS1-Syt1 interaction is observed in 9 month old Tg2576 AD mice without synaptic loss and before the appearance of amyloid plaque pathology (unpublished data). Together with other factors, the impaired PS1-Syt1 binding may weaken the synapse via local increase in the Aβ42/40 ratio and deficits in the overall protein exocytosis/neurotransmitter release, contributing to the synaptic loss.

Finally, since reduced Syt1 level and diminished PS1-Syt1 binding have a detrimental effect on PS1 conformation, we reasoned that overexpression of Syt1 in vivo in the brain might induce the protective “open” PS1 subdomain architecture. Indeed, we found that AAV-mediated delivery of the Syt1 into mouse hippocampi promotes the PS1-Syt1 binding, and impels the PS1 protective “open” conformational state. These findings suggest a therapeutic potential through fostering the PS1-Syt1 interaction for AD treatment. It is possible that this strategy may constitute a synapse-specific complementary approach to using GSMs. GSMs are known to reduce the pathogenic Aβ42/40 ratio [78–80], and a number of GSMs tested had promoted “open” PS1 conformation in both neuronal and non-neuronal cells [21, 22, 81].

Collectively, the data reveal novel role of PS1 and Syt1 at the synapse; present a reduction in the PS1-Syt1 interactions as potential molecular contributor to AD pathogenesis; and open avenues for novel synapse-specific AD-targeting therapeutic interventions.

## Conclusions

The study reveals novel role of presenilin 1 (PS1) and synaptotagmin 1 (Syt1) at the synapse. It demonstrates that PS1 and Syt1 can influence each other function via direct interaction. Furthermore, it reports that the PS1-Syt1 binding is reduced in sporadic Alzheimer’s disease (sAD) brains, which may contribute to the abnormal PS1/γ-secretase conformation, increased amyloid β (Aβ) 42/40 ratio, and impaired neurotransmitter release. The findings in this study indicate that fostering PS1-Syt1 interaction might serve as a novel synapse-specific therapeutic approach for AD. In summary, the reported data point towards novel mechanism regulating synaptic physiology and relative generation of the longer to shorter Aβ species, describe new pathogenic mechanisms of AD, and propose novel therapeutic target for the disease.

## Additional files

Additional file 1: Fluorescence lifetime and corresponding FRET efficiency values recorded in the FLIM assays. The table summarizes the mean ± SEM fluorescence lifetimes and corresponding, calculated FRET efficiencies for the all presented FLIM analyses. (XLSX 14 kb)
Additional file 2: Inhibition of PS1-Syt1 interaction does not affect APP expression and APP-CTF levels. The representative western blot demonstrates levels of APP-FL, APP-CTFs and Gapdh, as a loading control, in the protein lysates extracted from primary neurons pre-treated with scramble or PS1-LNT peptides for 2 h and stimulated for 15 min with 50 mM KCl. The adjacent graph shows the quantification of the APP-FL levels relative to Gapdh, and APP processing rate as an APP-CTFs/APP-FL ratio. The values were normalized to the scramble pre-treated cells. The data are presented as mean ± SEM, *n* = 10-11. Statistical significance was determined using two-tailed unpaired Student’s t-test, ns *p* > 0.05. (TIF 3734 kb)
Additional file 3: Inhibition of the PS1-Syt1 interaction leads to intraneuronal Aβ accumulation. The representative images demonstrate immunofluorescent staining of primary neurons pre-treated with scramble or PS1-LNT peptides, and depolarized for 15 min with 50 mM KCl. Anti-MAP2 (red) and anti-β-amyloid 17–24, clone 4G8 (green) antibodies were used; scale bar 10 μm. The adjacent graph presents the quantification of the fluorescent intensity of Aβ/APP normalized to the MAP2 fluorescence. The values were normalized to the scramble treated cells. The data are presented as mean ± SEM, *n* = 105 for scramble and *n* = 96 for PS1-LNT, *n* = number of neurons analyzed in 4 independent experiments. Statistical significance was determined using two-tailed unpaired Student’s t-test, ****p* < 0.001. (TIF 5598 kb)

## Abbreviations

Aβ: Amyloid β
APP: Amyloid precursor protein
fAD: Familial Alzheimer’s disease
FLIM: Fluorescence lifetime imaging microscopy
FRAP: Fluorescence recovery after photobleaching
FRET: Förster resonance energy transfer
PS1: Presenilin 1
sAD: Sporadic Alzheimer’s disease
SypHy: Synaptophluorin; fluorescent reporter of exocytosis
Syt1: Synaptotagmin 1
wt: Wild type
