# Supplementary figures and images for: Dynamic presenilin 1 and synaptotagmin 1 interaction modulates exocytosis and amyloid β production
Source: Mol Neurodegener. 2017 Feb 13;12:15. doi: 10.1186/s13024-017-0159-y (PMC5307796; doi:10.1186/s13024-017-0159-y)

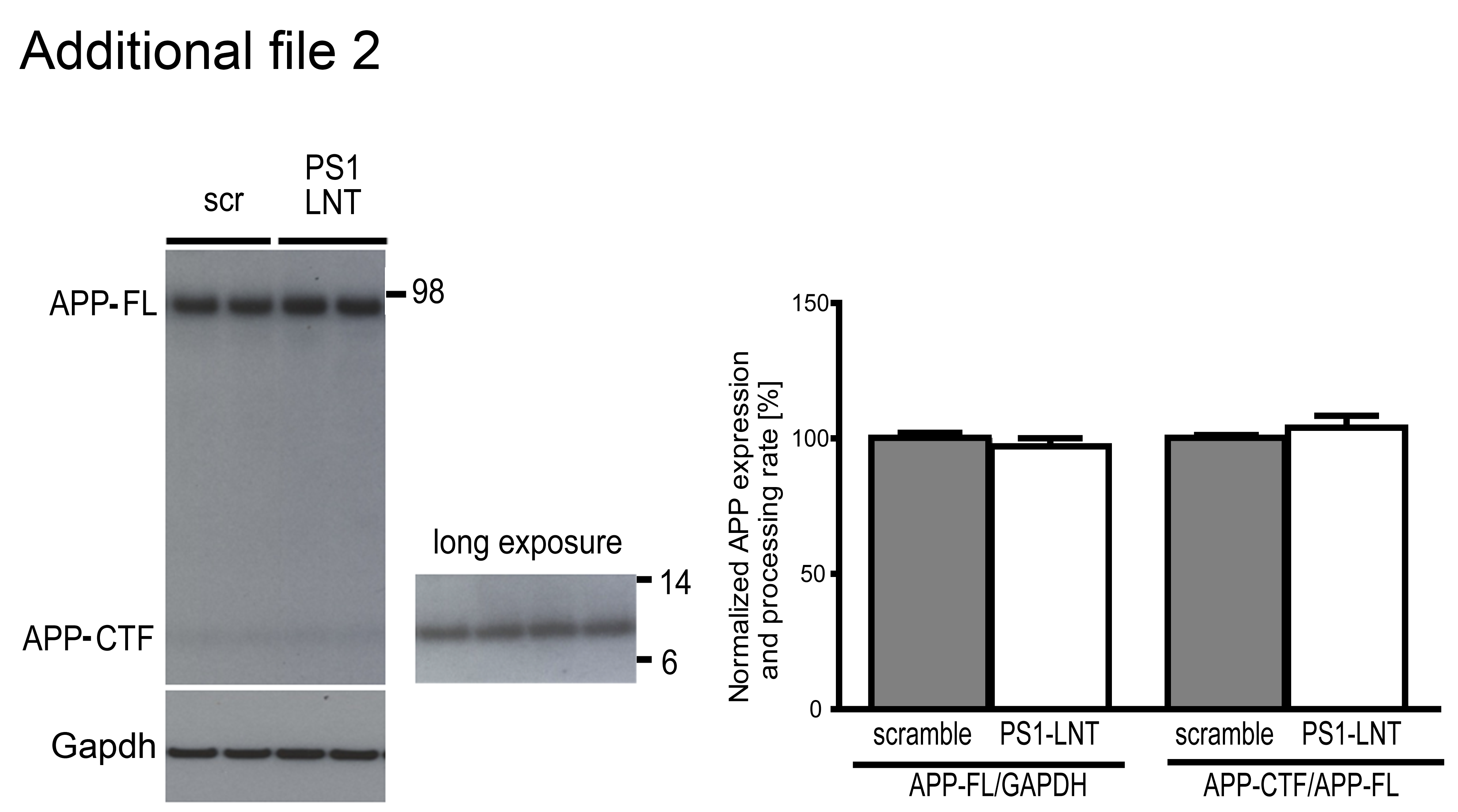

Supplement: Additional file 2: — Inhibition of PS1-Syt1 interaction does not affect APP expression and APP-CTF levels. The representative western blot demonstrates levels of APP-FL, APP-CTFs and Gapdh, as a loading control, in the protein lysates extracted from primary neurons pre-treated with scramble or PS1-LNT peptides for 2 h and stimulated for 15 min with 50 mM KCl. The adjacent graph shows the quantification of the APP-FL levels relative to Gapdh, and APP processing rate as an APP-CTFs/APP-FL ratio. The values were normalized to the scramble pre-treated cells. The data are presented as mean ± SEM, n = 10-11. Statistical significance was determined using two-tailed unpaired Student’s t-test, ns p > 0.05. (TIF 3734 kb) [file 13024_2017_159_MOESM2_ESM.tif]

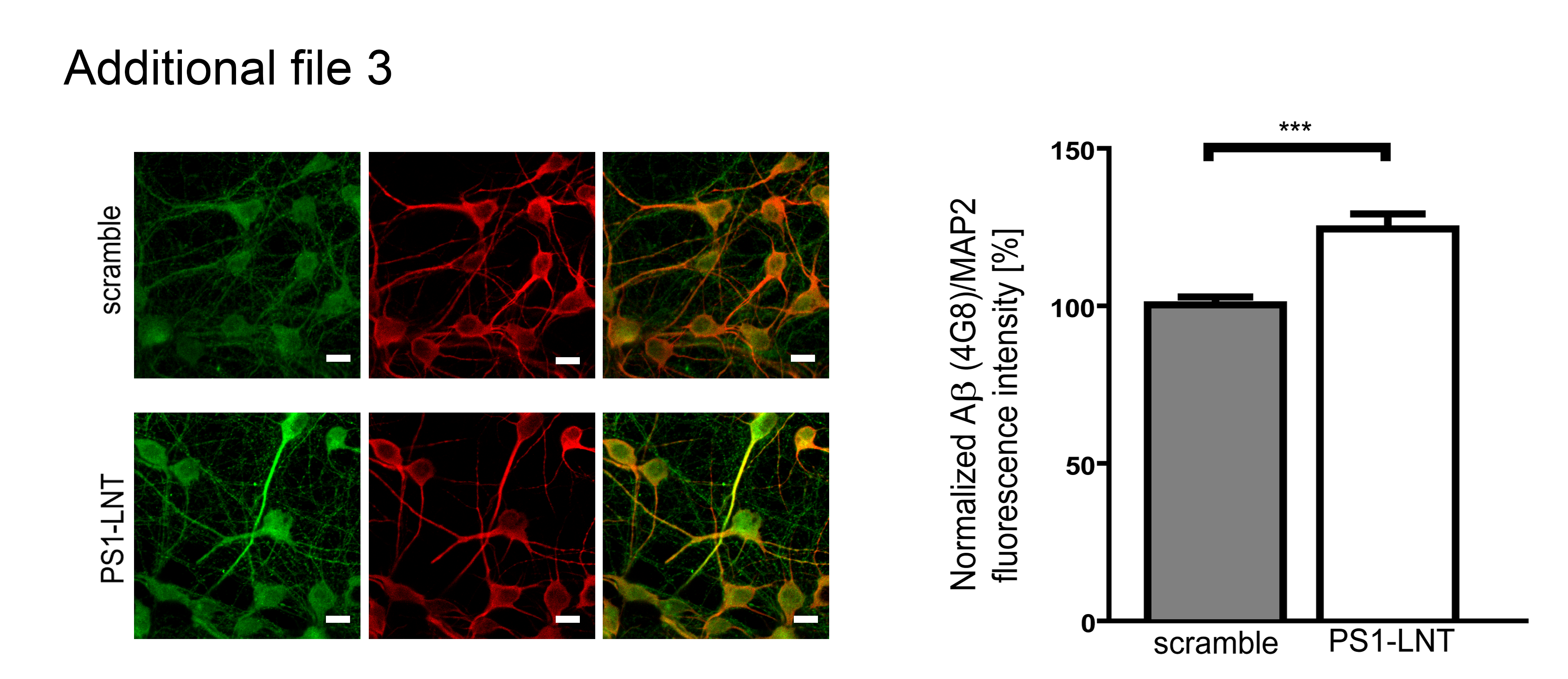

Supplement: Additional file 3: — Inhibition of the PS1-Syt1 interaction leads to intraneuronal Aβ accumulation. The representative images demonstrate immunofluorescent staining of primary neurons pre-treated with scramble or PS1-LNT peptides, and depolarized for 15 min with 50 mM KCl. Anti-MAP2 (red) and anti-β-amyloid 17–24, clone 4G8 (green) antibodies were used; scale bar 10 μm. The adjacent graph presents the quantification of the fluorescent intensity of Aβ/APP normalized to the MAP2 fluorescence. The values were normalized to the scramble treated cells. The data are presented as mean ± SEM, n = 105 for scramble and n = 96 for PS1-LNT, n = number of neurons analyzed in 4 independent experiments. Statistical significance was determined using two-tailed unpaired Student’s t-test, ***p < 0.001. (TIF 5598 kb) [file 13024_2017_159_MOESM3_ESM.tif]
